# TRIM5α Restricts Flavivirus Replication by Targeting the Viral Protease for Proteasomal Degradation

**DOI:** 10.1016/j.celrep.2019.05.040

**Published:** 2019-06-11

**Authors:** Abhilash I. Chiramel, Nicholas R. Meyerson, Kristin L. McNally, Rebecca M. Broeckel, Vanessa R. Montoya, Omayra Méndez-Solís, Shelly J. Robertson, Gail L. Sturdevant, Kirk J. Lubick, Vinod Nair, Brian H. Youseff, Robin M. Ireland, Catharine M. Bosio, Kyusik Kim, Jeremy Luban, Vanessa M. Hirsch, R. Travis Taylor, Fadila Bouamr, Sara L. Sawyer, Sonja M. Best

**Affiliations:** 1 Innate Immunity and Pathogenesis Section, Laboratory of Virology, Rocky Mountain Laboratories (RML), National Institute of Allergy and Infectious Diseases (NIAID), National Institutes of Health (NIH), Hamilton, MT 59840, USA; 2 Department of Molecular, Cellular and Developmental Biology, University of Colorado Boulder, Boulder, CO 80309, USA; 3 Research Technology Branch, RML, NIAID, NIH, Hamilton, MT 59840, USA; 4 Department of Medical Microbiology and Immunology, College of Medicine and Life Sciences, University of Toledo Health Science Campus, Toledo, OH 43606, USA; 5 Immunity to Pulmonary Pathogens Section, Laboratory of Bacteriology, RML, NIAID, NIH, Hamilton, MT 59840, USA; 6 Program in Molecular Medicine, University of Massachusetts Medical School, Worcester, MA 01655, USA; 7 Laboratory of Molecular Microbiology, NIAID, Bethesda, MD 20892, USA; 8 These authors contributed equally; 9 Lead Contact

## Abstract

Tripartite motif-containing protein 5α (TRIM5α) is a cellular antiviral restriction factor that prevents early events in retrovirus replication. The activity of TRIM5α is thought to be limited to retroviruses as a result of highly specific interactions with capsid lattices. In contrast to this current understanding, we show that both human and rhesus macaque TRIM5α suppress replication of specific flaviviruses. Multiple viruses in the tick-borne encephalitis complex are sensitive to TRIM5α-dependent restriction, but mosquito-borne flaviviruses, including yellow fever, dengue, and Zika viruses, are resistant. TRIM5α suppresses replication by binding to the viral protease NS2B/3 to promote its K48-linked ubiquitination and proteasomal degradation. Importantly, TRIM5α contributes to the antiviral function of IFN-I against sensitive flaviviruses in human cells. Thus, TRIM5α possesses remarkable plasticity in the recognition of diverse virus families, with the potential to influence human susceptibility to emerging flaviviruses of global concern.

## In Brief

The antiviral activity of TRIM5α is thought to be limited to retroviruses as a result of highly specific interactions with capsid lattices. Here, Chiramel et al. demonstrate that TRIM5α restricts replication of specific flaviviruses by binding and degrading the viral protease.

## Graphical Abstract

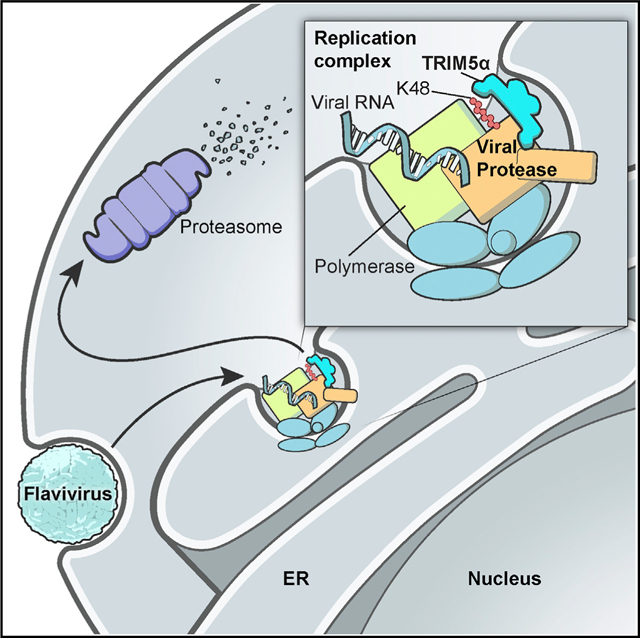

## INTRODUCTION

Flaviviruses (family *Flaviviridae*) include 53 recognized virus species, of which 40 are known to cause disease in humans, with over 40% of the world’s population at risk of flavivirus infection annually (Brady et al., 2012). These viruses have a high potential for emergence into human populations, as witnessed historically through global emergence of dengue virus (DENV), West Nile virus (WNV), and Zika virus (ZIKV). Additional (re)emerging viruses of considerable medical importance include yellow fever virus (YFV), Japanese encephalitis virus (JEV), and members of the tick-borne encephalitis virus (TBEV) serogroup. Flaviviruses share in common a positive-sense single-stranded RNA (ssRNA) genome encoding a single polyprotein that is cleaved by host cell signalases (Zhang et al., 2016) and the viral protease to generate three structural (capsid [C], pre-membrane [M], and envelope [E]) and seven nonstructural (NS) proteins (NS1, NS2A, NS2B, NS3, NS4A, NS4B, and NS5) (Lindenbach et al., 2007). Two of the NS proteins have enzymatic activity; the NS3 protein encodes the viral RNA helicase and together with its co-factor NS2B (NS2B/3) functions as the viral protease, whereas NS5 possesses both methyltransferase (MTase) and RNA-dependent RNA polymerase (RdRP) activities.

The repeated emergence of flaviviruses as human pathogens is in part due to the fact that they are arthropod-borne, transmitted by mosquitoes and ticks. In addition, the zoonotic reservoir species supporting virus replication in nature are highly diverse. For example, small mammals, particularly rodents, are thought critical for the maintenance of transmission cycles of TBEV and related viruses. In contrast, WNV utilizes birds, whereas DENV, ZIKV, and YFV evolved in non-human primates before at least DENV and ZIKV established urban transmission cycles maintained exclusively through human infections (Vasilakis and Weaver, 2017). The ability of a virus to avoid or evade host antiviral responses is essential to establish replication and transmission (Versteeg and García-Sastre, 2010). However, it is not fully understood how evolution in different reservoir hosts to avoid innate immunity has shaped replication and pathogenesis of different flaviviruses following infection of humans. Host-specific interactions with the interferon (IFN) response have been demonstrated for DENV and ZIKV that can only antagonize IFN-dependent signaling in the context of primate hosts (Best, 2017; Stabell et al., 2018). However, the IFN-stimulated genes (IGSs) that might also contribute to host-specific restriction of flaviviruses are not well characterized.

Tripartite motif-containing proteins (TRIMs) are strong candidates for mediating host-specific restriction of virus replication in the context of an IFN response. Approximately 100 tripartite TRIMs exist in the human genome (Han et al., 2011), many of which are ISGs with functions as direct antiviral restriction factors or as modulators of the cellular response to infection (Rajsbaum et al., 2014). The most characterized primate TRIM is TRIM5α, which functions as a cellular antiviral restriction factor with exquisite specificity, thought to restrict only retroviruses through complex interactions with the capsid lattice structure that accelerates uncoating of the viral nucleic acid and also blocks reverse transcription (Ganser-Pornillos et al., 2011; Stremlau et al., 2006; Wu et al., 2013). The significant influence of TRIM5α is exemplified by the observations that its antiviral activity drives lentivirus evolution (Wu et al., 2013) and limits cross-primate species transmission (Hatziioannou et al., 2006). Importantly, the relative ability of TRIM5α to bind retrovirus capsid lattices directly impacts primate species susceptibility to infection. For example, TRIM5α from Old World monkeys, such as rhesus macaques (rhTRIM5α), exerts potent antiviral activity against HIV-1 to confer host resistance. In contrast, human TRIM5α (hTRIM5α) only weakly interacts with HIV-1 capsid lattices, and this reduced efficacy may promote HIV-1 transmission and disease progression (Merindol and Berthoux, 2015). The antiviral specificity of TRIM5α has evolved rapidly in the past 30 million years of primate evolution, with particularly strong signatures of positive selection over the last 4–5 million years (Kaiser et al., 2007; Sawyer et al., 2005). Evolutionary studies support the conclusion that TRIM5α positive selection throughout primate evolution is driven at the interaction interface between TRIM5α and retrovirus capsids and, thus, reinforce the paradigm that the antiviral activity of TRIM5α and its role in host resistance is specific to the retroviruses (McCarthy et al., 2015).

Given the extensive evolution of multiple medically important flaviviruses with primate species, we examined the antiviral capacity of both rhTRIM5α and hTRIM5α toward the vector-borne flaviviruses. Surprisingly, both rhTRIM5α and hTRIM5α possessed potent antiviral function against specific flaviviruses within the TBEV serogroup but not toward mosquito-borne flaviviruses. The antiviral activity of TRIM5α was mediated through interactions with the viral protease NS2B/3 at sites of virus replication, and association of TRIM5α with NS2B/3 from a sensitive virus resulted in proteasomal degradation of the viral protein. Importantly, hTRIM5α contributed significantly to the antiviral effects of type I IFN against sensitive tick-borne viruses. However, TRIM5α was ineffective against important mosquito-borne flaviviruses, including YFV, DENV, and ZIKV. Thus, this work reveals an unexpected role for primate TRIM5α as an anti-flavivirus restriction factor that may influence human susceptibility to infection.

## RESULTS

### TRIM5α Is a Functional Restriction Factor for Flaviviruses

**Figure 1.**
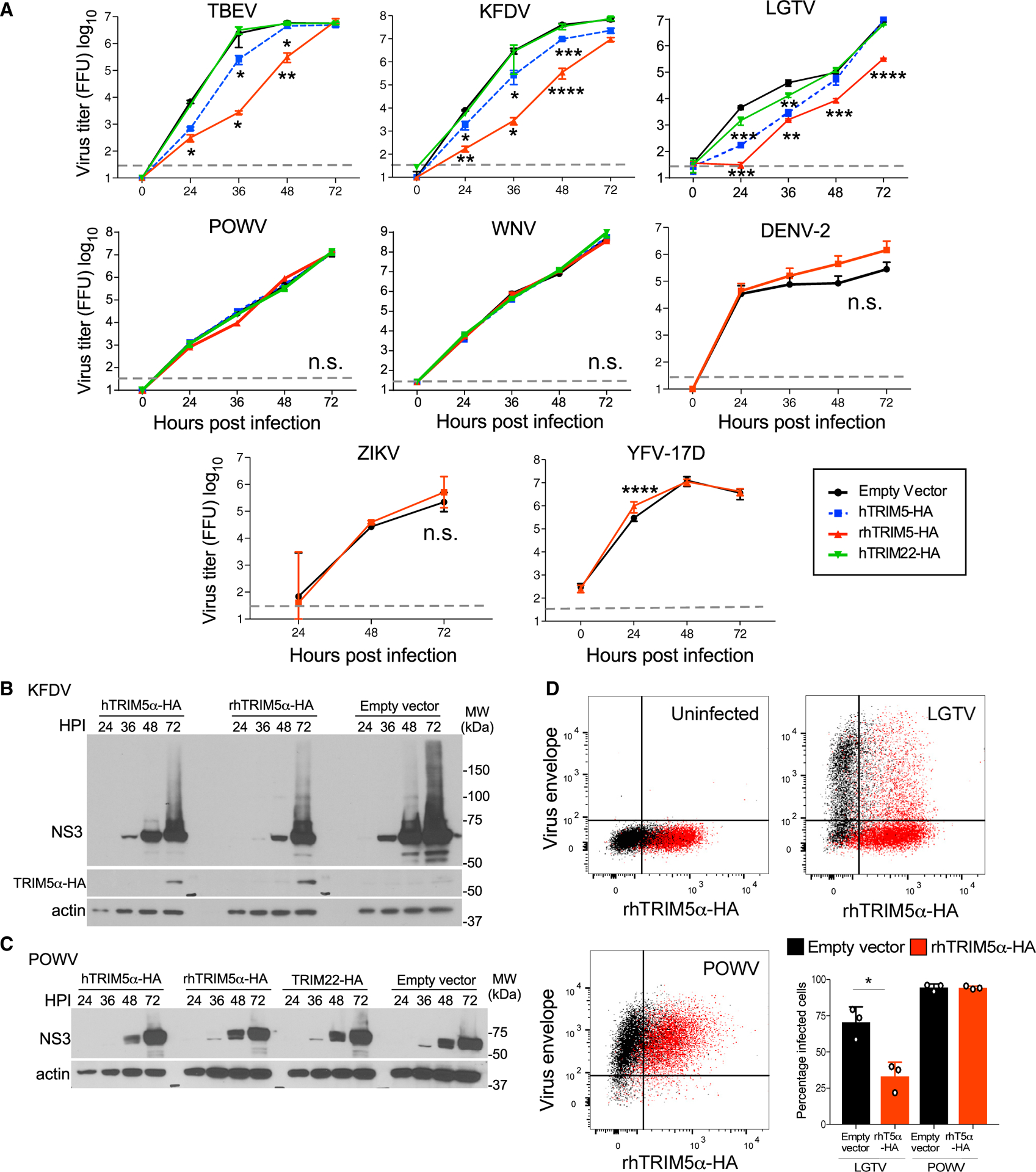
Stable Expression of TRIM5α in HEK293 Cells Restricts Replication of Specific Flaviviruses (A) HEK293 cells stably overexpressing human (h) or rhesus (rh) TRIM5α-HA, hTRIM22-HA, or empty vector (control) were infected with tick-borne encephalitis virus (TBEV), Kyasanur Forest disease virus (KFDV), Langat virus (LGTV), Powassan virus (POWV), West Nile virus (WNV), dengue virus (DENV-2), Zika virus (ZIKV), or yellow fever virus (YFV) with a multiplicity of infection (MOI) of 0.001 (except YFV at MOI 0.1). Infectious virus release was determined in supernatants by plaque assay. All data are from 3 independent experiments performed in triplicate (mean ± SD, *p < 0.05, **p < 0.01, ***p < 0.001, ****p < 0.0001; n.s., not significant, by Mann-Whitney). Grey dotted line indicates limit of detection. (B and C) NS3 protein levels in stable HEK293 cells infected with (B) KFDV or (C) POWV. The western blots are generally representative of 2 or 3 experiments, unless otherwise stated. (D) Dot plots depicting an overlay of E protein in empty vector (black) or rhTRIM5α-HA cells (red) infected with LGTV or POWV measured by flow cytometry. The percentage of cells infected as measured by E protein staining is quantified in the bar graphs (mean ± SD, data from 3 independent experiments in triplicate, *p < 0.05, one-way ANOVA with Sidak post-test). See also Figure S1.

The association of various mosquito-borne flaviviruses with primates prompted us to test whether ectopic expression of TRIM5α might have anti-flavivirus activity. HEK293 cells were engineered to stably express various TRIM5α proteins (Figure S1A). The expression of rhTRIM5α restricted infection of vesicular stomatitis virus glycoprotein (VSV-G) pseudotyped HIV-1 in 293 cells, demonstrating that these cells are appropriate to observe TRIM5-mediated restriction (Figure S1B). Compared to empty vector control cells, expression of either hTRIM5α or rhTRIM5α restricted replication of related viruses in the TBEV serogroup, including TBEV (strain Sofjin), Kyasanur Forest disease virus (KFDV), and Langat virus (LGTV; an attenuated member of the TBEV serocomplex) but not WNV (strain NY99), DENV (strain NGC, serotype 2), ZIKV (strain 2013 French Polynesia), or YFV (strain 17D) (Figure 1A). TRIM5α did not affect replication of Powassan virus (POWV; strain LB) despite this virus also belonging to the TBEV serogroup. The impact of hTRIM5α or rhTRIM5α on replication of sensitive flaviviruses was significant, reducing the production of infectious virus by up to 1,000-fold during the exponential phase of virus growth. hTRIM5α was functional but less efficient, imposing a 90% reduction, but this may be attributable to the lower expression of hTRIM5α compared to rhTRIM5α (Figure S1A). Therefore, we also used CrFK cells stably expressing hTRIM5α-hemagglutinin (HA) as a cell model historically used to examine retrovirus restriction as they lack intrinsic TRIM5α expression (Zhang et al., 2006). Expression of hTRIM5α suppressed replication of both TBEV (Figure S1C) and LGTV (an attenuated member of the TBEV serocomplex) (data not shown) but not WNV (Figure S1D). In HEK293 cells that support more optimal flavivirus growth, restriction was observable up to a starting multiplicity of infection (MOI) of 10 (Figure S1E), but replication of TRIM5α-sensitive viruses eventually overcame restriction, which is consistent with viral saturation of antiviral restriction factors (Cowan et al., 2002; Figure 1A). A related hTRIM with anti-retrovirus function, TRIM22 (Tissot and Mechti, 1995), did not impact replication of TBEV, KFDV, or LGTV, demonstrating a specific role for TRIM5α in flavivirus restriction (Figure 1A). Suppressed replication of KFDV was also observed at the level of protein expression, with reduced accumulation of NS3 in cells expressing hTRIM5α-HA or rhTRIM5α-HA compared to the empty vector controls (Figure 1B). Expression of the E protein of sensitive viruses was also reduced when examined by flow cytometry (Figure 1D). However, no reduction in either NS3 by western blot or E expression by flow cytometry was observed following POWV infection, supporting flavivirus-species-specific restriction by TRIM5α (Figures 1C and 1D).

**Figure 2.**
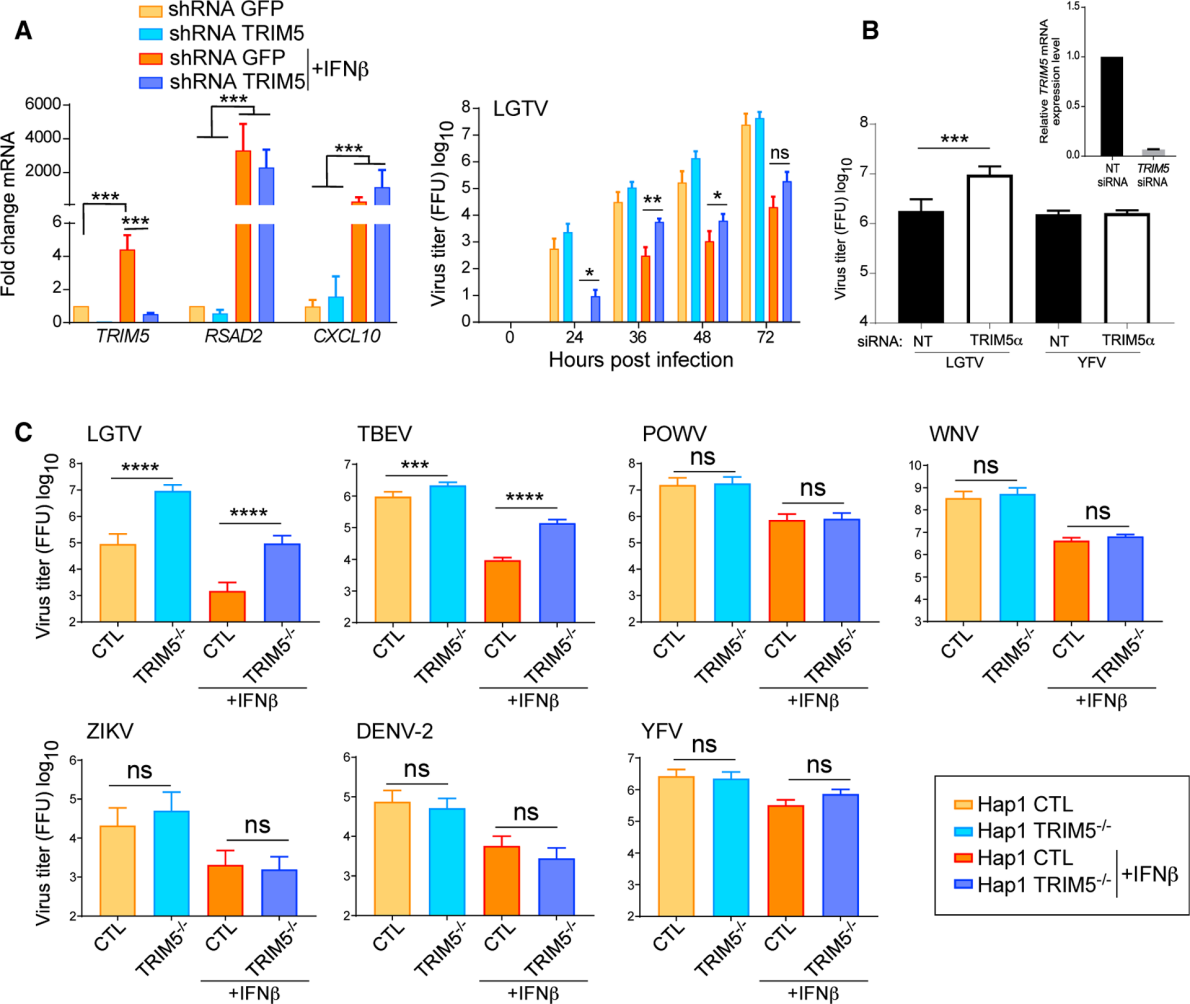
Endogenous hTRIM5α Is an ISG Required for the Antiviral Effects of IFNβ against TBEV and LGTV (A) The left panel shows qRT-PCR for *TRIM5*, *RSAD2*, or *CXCL10* mRNA isolated from A549 cells following transduction with lentiviruses expressing short hairpin RNAs (shRNAs) for GFP (control) or *TRIM5*, and untreated or treated with IFNβ (IFN) at 1000 U/ml for 6 h. The right panel shows LGTV titers in A549 cells that were left untreated or pre-treated with IFNβ for 6 h and infected at an MOI of 0.001. Supernatants were collected at the indicated times and titrated by plaque assay. All data are from 3 independent experiments performed in triplicate (mean ± SD, *p < 0.05, **p < 0.01 by Mann-Whitney; ns, not significant). (B) A549 cells were transfected with siRNAs specific for *TRIM5* or a non-targeting (NT) control. Cells were infected with LGTV or YFV at 48 h post-transfection (MOI, 0.001), and supernatants harvested for virus titration 48 h later. Data are from 3 independent experiments (mean ± SD, ***p < 0.001 by unpaired t test one-tailed). Inset shows the relative *TRIM5* mRNA expression measured by qRT-PCR in A549 cells. (C) Replication of LGTV, TBEV, POWV, WNV, ZIKV, DENV-2, and YFV (all infected at MOI 0.1) in Hap1 cells with *TRIM5* gene disruption by CRISPR/Cas9. Hap1 cells were left untreated or pretreated for 6 h with IFNβ. Data are from 2–3 independent experiments performed in triplicate (mean ± SD, ***p < 0.001, ****p < 0.0001 by one-way ANOVA with Tukey’s multiple comparisons post-test; ns, not significant).

To determine if hTRIM5α is a functional restriction factor, *TRIM5* mRNA was depleted by RNAi in A549 cells using lentivirus-delivered short hairpin RNA (shRNA), or *TRIM5* was knocked out using CRISPR/Cas9 in Hap1 cells. Cells were left untreated or treated with IFNβ for 6 h prior to infection to upregulate *TRIM5* expression and induce an antiviral state. Reduced *TRIM5* expression did not affect the responsiveness of cells to IFNβ as measured by upregulation of canonical ISGs, *RSAD2* (viperin), and *CXCL10* (Figure 2A). However, depletion of *TRIM5* partially relieved the antiviral effect of IFNβ on LGTV (Figure 2A). Transfection of A549 cells with an independent small interfering (siRNA) sequence targeted toward hTRIM5α also increased replication of LGTV but not YFV (Figure 2B). Furthermore, deletion of TRIM5 using CRISPR/Cas9 in Hap1 cells (Figures S1F and S1G) rescued ~2 log_10_ LGTV replication in the presence of IFNβ (Figure 2C). Virus replication was also increased for TBEV but not POWV, WNV, ZIKV, DENV, or YFV (Figure 2C). Together, these results identify TRIM5α as a restriction factor for specific species of flaviviruses and demonstrate that TRIM5α is an effector of the human type I IFN response to these viruses.

### TRIM5α Expression Restricts Viral RNA Replication

**Figure 3.**
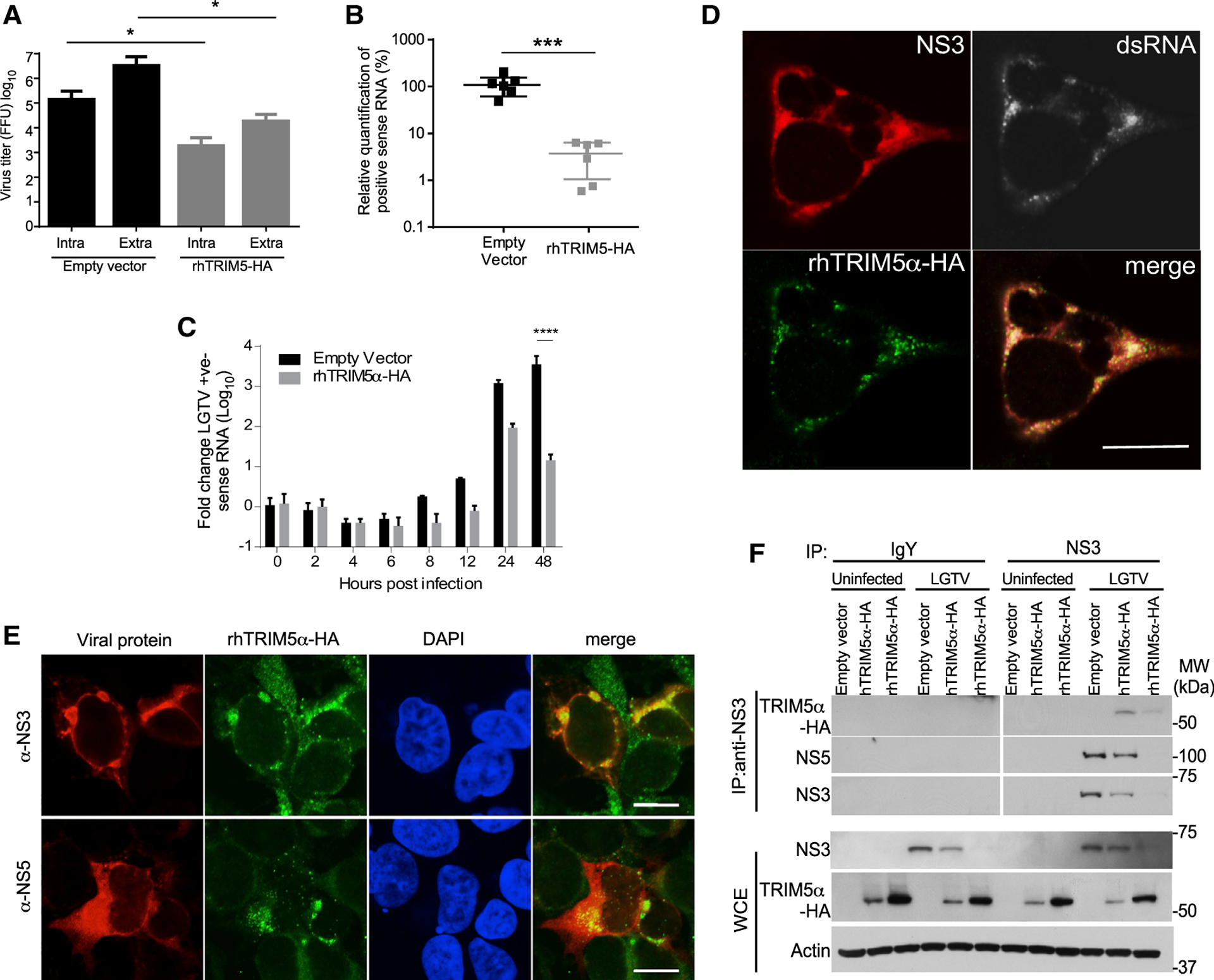
TRIM5α Restricts Flavivirus RNA Replication and Co-precipitates with the Viral Protease NS2B/3 (A) HEK293 cells with stable expression of rhTRIM5α-HA or the empty vector (control) were infected with LGTV (MOI of 0.01). Infectious virus in cell supernatants or intracellular virus was quantified by plaque assay at 48 hpi (mean ± SD, data from 3 independent experiments in triplicate, *p < 0.05 one-tailed, Mann-Whitney). (B) Accumulation of LGTV positive-sense viral RNA in cells infected in (A) was determined at 48 hpi by qRT-PCR (mean ± SD, *p < 0.05; ***p < 0.001, unpaired t test from 3 independent experiments in triplicate). (C) Changes in genomic RNA over time following binding of LGTV to control and rhTRIM5α-HA-expressing HEK293 cells at 4°C and three washes with DPBS (mean ± SD, ****p < 0.0001, 2-way ANOVA with Sidak’s post-test from 3 independent experiments in triplicate). (D) Colocalization of NS3 (red), dsRNA (greyscale), and rhTRIM5α (green) in HEK293 rhTRIM5α-HA LGTV-infected cells at 24 hpi by IFA (MOI of 5). Scale bar, 10 μm. (E) Colocalization of NS3 (red) or NS5 (red) and rhTRIM5α (green) in HEK293 rhTRIM5α-HA LGTV-infected cells at 24 hpi by IFA. Nuclei are counterstained with 4′,6-diamidino-2-phenylindole (DAPI; blue) (MOI of 5). Scale bar, 10 μm. (F) Interactions between rhTRIM5α or hTRIM5α with NS3 at 48 hpi with LGTV shown by immunoprecipitation (IP) of NS3 from infected HEK293 cells. WCE, whole-cell extract. See also Figure S2.

**Figure 4.**
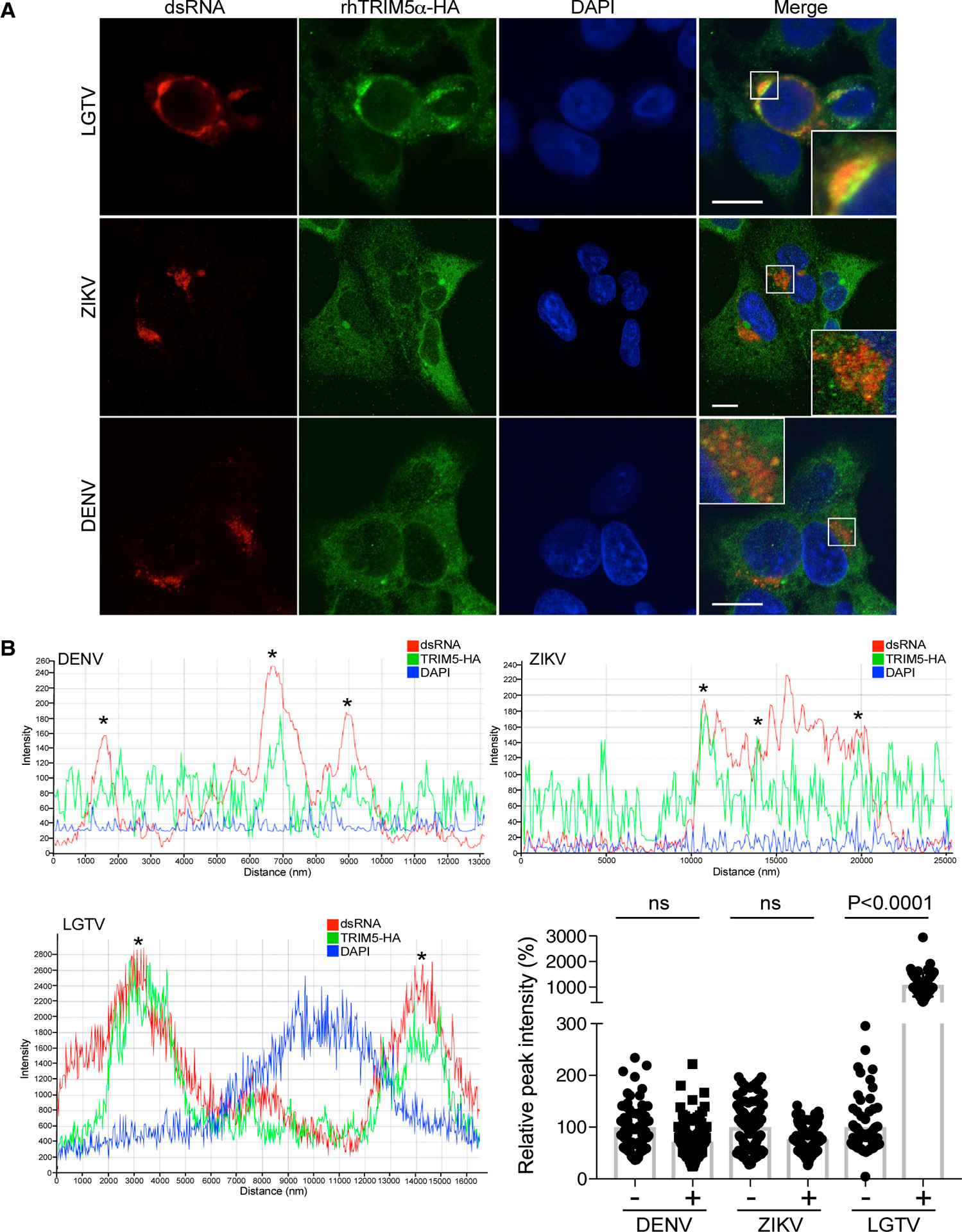
rhTRIM5α Does Not Form Cytoplasmic Bodies at Sites of dsRNA during Replication of ZIKV or DENV (A) Stable rhTRIM5α-HA cells were infected with LGTV (MOI 5), ZIKV (MOI 0.01), or DENV (MOI 0.01) and stained for dsRNA (red) and rhTRIM5α (green) at 24 hpi. Nuclei were counterstained with DAPI. Scale bar, 10 μm. Insets show the region indicated by a white box. (B) Examples of the intensity profiles along vectors drawn through dsRNA staining in 3 fields of rhTRIM5α-HA cells infected with LGTV, ZIKV, or DENV (mean ± SD, ****p < 0.0001, 2-way ANOVA with Sidak’s post-test). Asterisks indicate colocalization between rhTRIM5α-HA and concentrations of dsRNA as measured by intensity along vectors.

To determine which step in the flavivirus life cycle was restricted by TRIM5α, LGTV replication was examined in rhTRIM5α-HA HEK293 cells. At 48 h post-infection (hpi), supernatants and cell lysates were subjected to three cycles of freeze-thaw lysis to compare levels of intra- and extracellular virus. In the presence of rhTRIM5α, no change in the ratio (~1:10) of intracellular: extracellular infectious virus was observed (Figure 3A), although intracellular accumulation of positive-sense (genomic) viral RNA was reduced by approximately 50-fold (Figure 3B). Viral entry was not affected, as differences in positive-sense RNA were not apparent after virus entry until at least 8–12 hpi when flavivirus RNA replication is initiated (Chu and Westaway, 1985; Lindenbach et al., 2007; Figure 3C). Thus, TRIM5α imposes a block in virus replication at or preceding RNA replication without affecting virus entry or release. In flavivirus-infected cells, cellular localization of double-stranded RNA (dsRNA) is an obligate marker of sites of replication, and most perinuclear foci containing NS3 (the viral protease and RNA helicase) also colocalize with dsRNA, suggesting these perinuclear foci are sites of active replication (Westaway et al., 1997; Figure S2A). In infected cells, small aggregates of rhTRIM5α often termed cytoplasmic bodies (Diaz-Griffero et al., 2006) colocalized with NS3 and dsRNA, suggesting that TRIM5α is recruited to replication complexes (Figures 3D). RhTRIM5α aggregates also colocalized with NS5 (the viral RdRP) but only at perinuclear sites likely together with NS3 at the endoplasmic reticulum (ER) (Figure 3E). Recruitment of hTRIM5α to sites of NS3 expression was also observed in LGTV-infected cells (Figure S2B). Areas of colocalization were observable between TRIM5α and dsRNA in the context of DENV or ZIKV, but infection did not induce strong aggregation of TRIM5α (Figures 4A and 4B). Next, we validated the association of either hTRIM5α or rhTRIM5α with NS3 by immunoprecipitation (IP) in LGTV-infected cells (Figure 3F). Despite low levels of viral protein associated with restriction, IP of NS3 from infected cells resulted in co-precipitation with either hTRIM5α or rhTRIM5α (Figure 3F). As expected, NS5 also co-precipitated with NS3 in infected cells, which supports the immunofluorescence assay (IFA) data and suggests that TRIM5α interactions with NS3 occur at sites of virus replication where NS3 and NS5 interact. Consistent with a lack of TRIM5α aggregation at sites of dsRNA staining (Figures S2C and S2D), IP of NS3 from WNV-infected cells did not result in co-precipitation of rhTRIM5α (Figure S2E). Thus, TRIM5α localizes to viral replication complexes and suppresses RNA replication in a flavivirus-specific manner.

### TRIM5α Targets the Flavivirus Protease for Proteasomal Degradation

**Figure 5.**
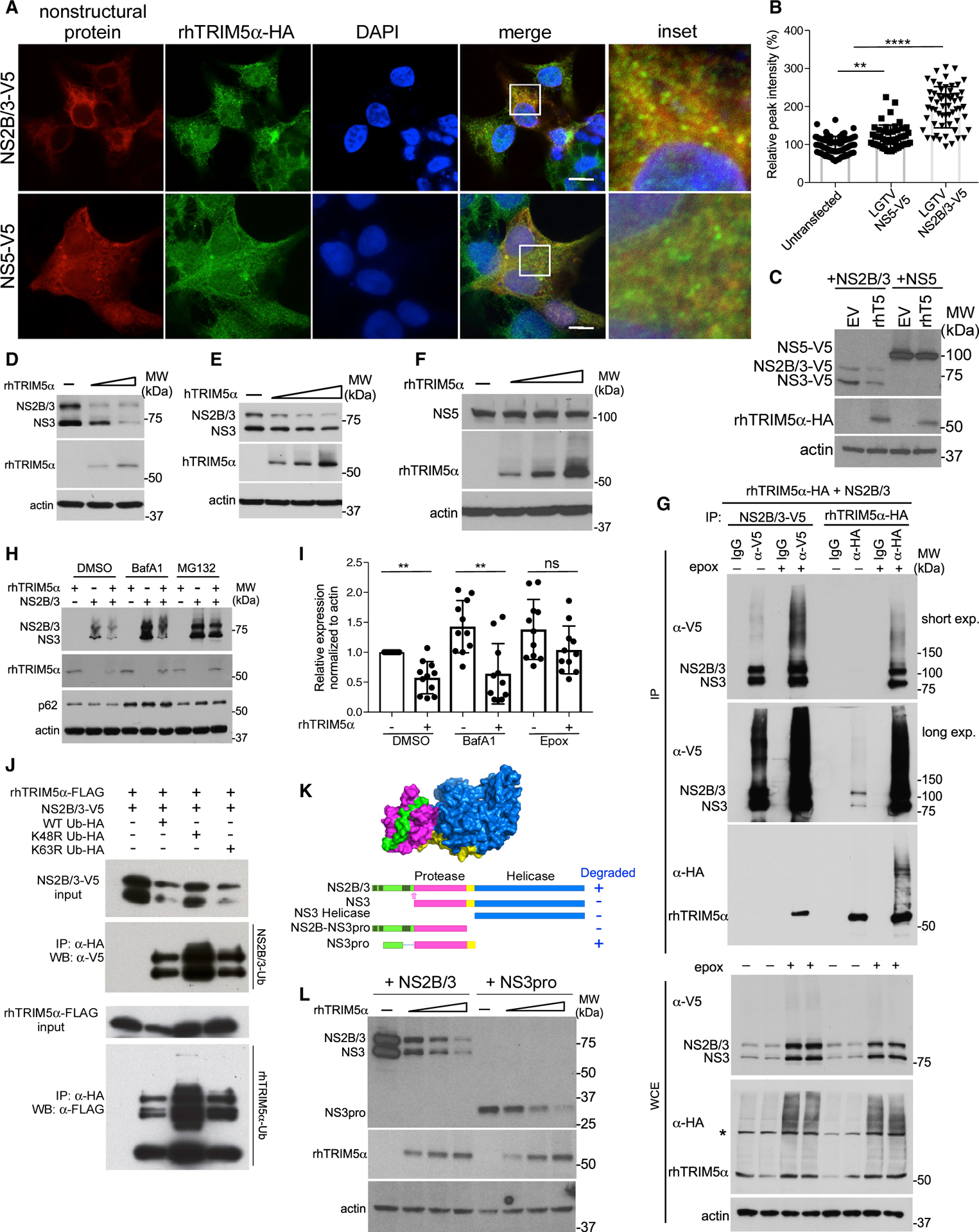
Binding of the Flavivirus Protease by rhTRIM5α Is Conformation-Dependent and Results in Proteasome-Dependent Degradation of NS2B/3 (A) Stable HEK293 rhTRIM5α-HA (green) cells were transfected with plasmids coding for either NS2B/3-V5 or NS5-V5 (red) from LGTV and imaged by confocal microscopy. Scale bar, 10 μm. (B) Relative intensity of TRIM5 aggregates were measured along vectors drawn in 3 fields of cells expressing LGTV NS2B/3 or NS5, with example vectors shown in Figure S3B (mean ± SD, ****p < 0.0001, 2-way ANOVA with Dunnett’s post-test). (C) Western blot of LGTV NS2B/3-V5 or NS5-V5 in stable rhTRIM5α-HA or control HEK293 cells. (D–F) Western blot analysis of HEK293 cells transfected with (D) increasing amounts of rhTRIM5α-HA and constant amounts of LGTV NS2B/3-V5, (E) increasing amounts of hTRIM5α-HA and constant amounts of LGTV NS2B/3-V5, and (F) increasing amounts of rhTRIM5α-HA and constant amounts of LGTV NS5-V5. (G) Reciprocal coIP of rhTRIM5α-HA and LGTV NS2B/3-V5 following cotransfection and 4 h treatment with epoxomicin (200 nM). The asterisk indicates a non-specific band. (H) Western blot of LGTV NS2B/3-V5, rhTRIM5α-HA, and endogenous p62 in HEK293 cells following 4 h treatment with DMSO (vehicle), Baf-A1 (200 nM), or epoxomicin (200 nM). (I) Quantification of LGTV NS2B/3 expression with or without rhTRIM5α and treated with Baf-A1 or epoxomicin from 11 individual experiments (mean ± SD, **p < 0.01, 2-way ANOVA with Sidak’s post-test). (J) LGTV NS2B/3-V5 and rhTRIM5α-FLAG were co-expressed with ubiquitin (Ub)-HA wild type (WT) or K48R or K63R mutants in HEK293 cells. Target proteins were immunoprecipitated using anti-V5 or anti-FLAG antibodies and blots probed with anti-HA to examine Ub conjugation. (K) Domain structure of flavivirus NS2B/3 (PDB: 2vbc) and schematic representation of truncation mutants. (L) Western blot analysis of HEK293 cells transfected with increasing amounts of rhTRIM5α-HA and constant amounts of LGTV NS3pro. Lysates were probed specifically for HA, V5 and b-actin. See also Figure S3 and S4.

To examine interactions with NS3 and NS5 separately, stable rhTRIMα-HA cells were transfected with plasmids encoding LGTV NS2B/3 or NS5. NS2B was included as it forms an integral structural component of the NS3 protease active site and trans-membrane domains within NS2B target NS3 to ER membranes, with NS2B/3 being an important antiviral drug target (Luo et al., 2015). NS5 showed some co-localization with rhTRIM5α (Figure 5A) and caused low levels of TRIM5α aggregation (Figure 5B) but did not co-precipitate (Figure S3A). However, NS2B/3 expression caused rhTRIM5α to aggregate into discrete cytoplasmic bodies (Figures 5A, 5B, and S3B) and co-localize reminiscent of that observed following virus infection, and LGTV NS2B/3 strongly associated with rhTRIM5α by co-precipitation (Figure 5G). In addition, expression levels of NS2B/3 were reduced in cells expressing rhTRIM5α compared to the control cell line, whereas NS5 levels were not strongly affected (Figure 5C). To further explore this observation, a constant level of LGTV NS2B/3 was expressed with increasing amounts of either rhTRIM5α or hTRIM5α by transfection of expression plasmids. In either case, expression of both unprocessed NS2B/3 and NS3 generated through autonomous cleavage was reduced in a dose-dependent fashion (Figures 5D and 5E), although this effect was quickly saturated. Again, the expression of LGTV NS5 was not affected by rhTRIM5α expression (Figure 5F).

In the context of HIV-1, TRIM5α utilizes the proteasome (MG132 sensitive) for capsid disruption but not for restriction (Kutluay et al., 2013) and may also use lysosomes following autophagy (BafA1-sensitive) to degrade the capsid (Mandell et al., 2014a; Ribeiro et al., 2016). Treatment of NS2B/3-expressing cells with BafA1 to inhibit lysosomal degradation increased the expression of NS2B/3 when expressed alone but did not rescue the relative loss of NS2B/3 in the presence of rhTRIM5 (Figures 5H and 5I). This was despite the BafA1-sensitive rescue of p62/SQSTM1, which is a reported co-factor to TRIM5-mediated retrovirus restriction (O’Connor et al., 2010; Figure 5H). Selective autophagy of the HIV-1 capsid by TRIM5α is also mediated by Beclin, ATG5, p62, GABARAP, and LC3 (Mandell et al., 2014a), but siRNA-mediated knock down of these genes did not significantly relieve LGTV restriction (Figures S4A–S4C). Finally, the C-type lectin langerin, but not DC-SIGN, was previously shown to be sufficient for autophagic degradation of the HIV-1 capsid by hTRIM5α (Ribeiro et al., 2016). However, although DC-SIGN augmented LGTV replication as expected in its role as a flavivirus attachment factor (Davis et al., 2006), langerin expression had no effect and did not further increase the restriction of LGTV in TRIM5α-expressing cells (Figure S4D), strongly suggesting that selective autophagy following virus entry or establishment of viral replication complexes is not the main mechanism of restriction. In contrast, treatment with epoxomicin (Figures 5H and 5I) recovered the majority of NS3 in the presence of rhTRIM5α, implicating proteasomal degradation of NS2B/3. This was supported by reciprocal IP of NS2B/3 ectopically co-expressed with rhTRIM5 in the presence of epoxomicin demonstrating (1) increased interactions between TRIM5α and both the uncleaved NS2B/3 precursor and the mature, autocleaved NS3 protein, and (2) increased ubiquitination of NS2B/3 co-precipitating with TRIM5α (Figure 5G). TRIM5α did not appear to affect protease activity, as autocleavage to produce NS3 measured by the ratio of NS2B/3:NS3 did not change in the presence of TRIM5α (Figure S4E). Furthermore, overexpression of K48R-HA ubiquitin (Ub) that cannot make K48-linked Ub chains, but not K63R-HA Ub, rescued expression of both NS2B/3 and rhTRIM5α (Figure 5J), further suggesting that NS2B/3 degradation involves K48-linked ubiquitination, which generally involves the proteasome.

To determine the domain of NS2B/3 recognized by TRIM5α, degradation assays were performed on various truncated NS2B/3 constructs (Figure 5K). NS3 expressed without NS2B (Figure S4F) or the NS3 helicase domain alone (Figure S4G) was not sufficient for TRIM5α-mediated degradation. A construct containing the entire NS2B protein fused to the NS3 protease domain (NS2B-NS3pro) was also not degraded, suggesting that NS2B alone is not sufficient as a target (Figure S4H). However, expression of NS3pro containing the 40 amino acids of NS2B required for NS3 protease activity in frame with a flexible glycine linker, the NS3 protease domain and the linker sequence between the NS3 protease and helicase domains, enabled degradation (Figure 5L). Thus, the target for TRIM5α degradation requires NS2B in addition to NS3 sequences (NS3pro). Recognition of NS2B/3 is, therefore, likely dependent on protease conformation but is independent of protease activity, as the S138A active site mutant of NS2B/3 was also degraded (Figure S4I).

### TRIM5α Interaction with the Flavivirus Protease Is Associated with Virus Restriction

**Figure 6.**
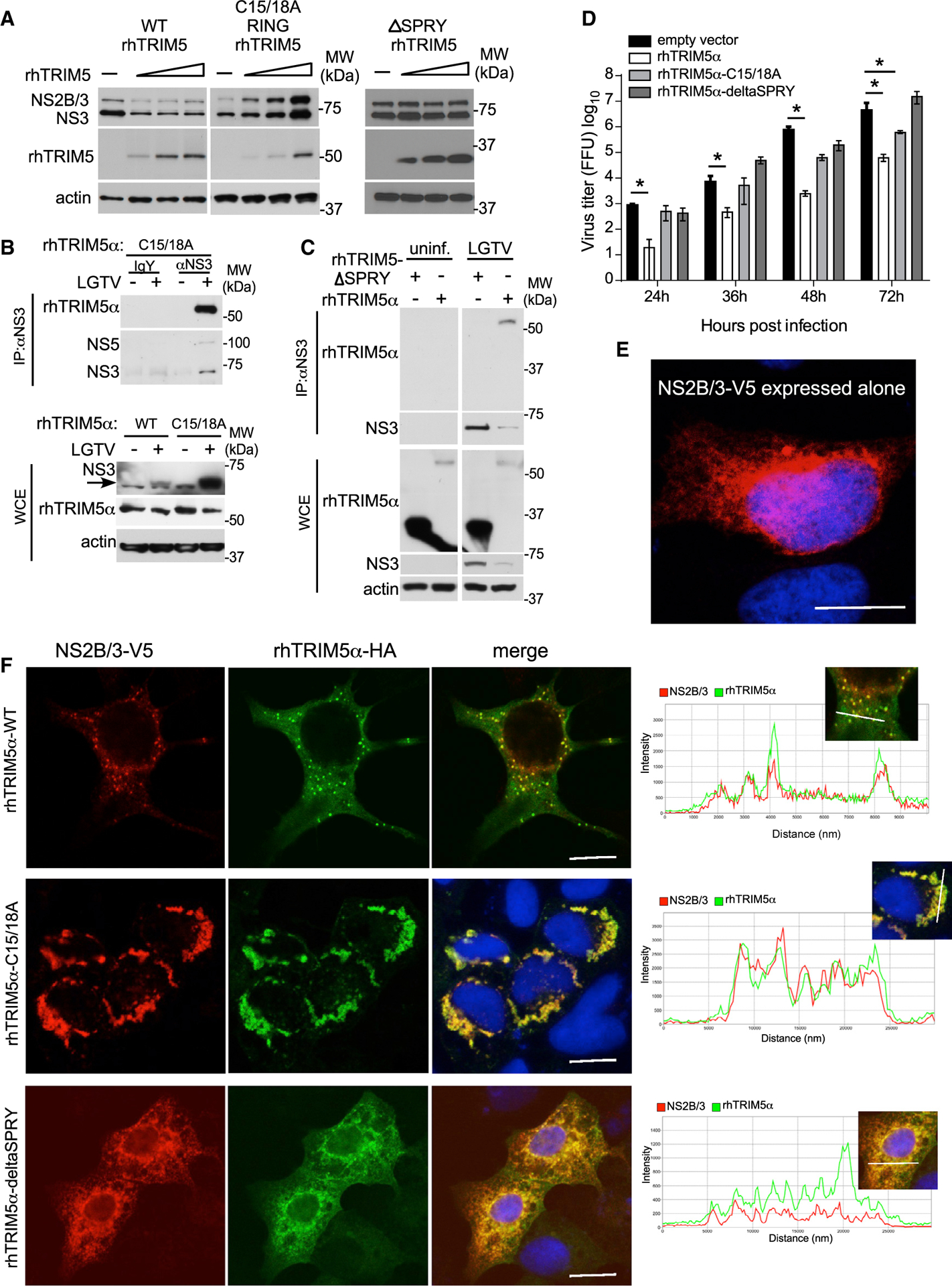
TRIM5α Interaction with the Flavivirus Protease Is Associated with Virus Restriction (A) Western blot analysis following transfection of constant amounts of LGTV NS2B/3-V5 plasmid with increasing amounts of rhTRIM5α-HA, RING mutant rhTRIM5(C15/C18A)-HA, or rhTRIM5-delta SPRY-HA, as indicated in HEK293 cells. (B and C) IP of NS3 from LGTV-infected HEK293 cells (MOI of 0.01; 48 hpi) stably expressing (B) RING rhTRIM5(C15/C18A)-HA or (C) rhTRIM5α-HA or rhTRIM5-delta SPRY-HA. (D) LGTV replication kinetics in HEK293 cells stably expressing rhTRIM5α-HA, RING mutant rhTRIM5(C15/C18A)-HA, rhTRIM5-delta SPRY, or the empty vector control following infection at MOI of 0.001. All data are from 3 independent experiments in triplicates (mean ± SD, *p < 0.05 Mann-Whitney test). (E and F) HEK293 cells were co-transfected with LGTV NS2B/3-V5 (shown expressed alone in E), WT rhTRIM5α-HA, RING mutant rhTRIM5(C15/C18A)-HA, or rhTRIM5-delta SPRY-HA (F). Slides were fixed and processed for indirect immunofluorescence staining with antibodies specific for HA (green) and V5 (red), and nuclei were counterstained with DAPI (blue). Images were analyzed using confocal microscopy with fluorescence intensity profiles measured across the white line of insets to demonstrate colocalization using Zen Imaging software. Scale bar, 10 μm. See also Figure S5.

The N terminus of TRIM proteins is composed of a RBCC motif, which includes a really interesting new gene (RING) domain, one or more B-box domains, and a coiled-coiled (CC) domain (Luban, 2012). The RING and B-box can mediate conjugation of Ub, thereby functioning as an E3 Ub ligase, whereas the CC domain allows oligomerization of TRIM proteins and formation of cytoplasmic bodies (Fletcher et al., 2018). The specificity of TRIM proteins is mainly determined by their C-terminal B30.2/SPRY domain that is responsible for binding to specific substrates, including retroviral capsids (Sawyer et al., 2005). The C15/18A RING mutant of rhTRIM5α did not degrade NS2B/3 (Figure 6A) and instead stabilized it consistent with retention of binding (Figure 6B). Restriction of infectious virus production was also dependent on rhTRIM5α RING function, particularly at early times post-infection (Figure 6D). Compared to co-expression of LGTV NS2B/3 with WT-rhTRIM5α-HA, the C15/18A RING mutant retained strong colocalization by IFA but lost the ability to form discrete cytoplasmic bodies (Figures 6E and 6F). In contrast, deletion of the B30.2/SPRY domain eliminated degradation of NS2B/3 (Figure 6A) associated with failure to bind NS3 in infected cells (Figure 6C), reduced colocalization with ectopically expressed NS2B/3 (Figure 6F), and the loss of antiviral activity (Figure 6D). Importantly, these data directly link TRIM5α binding and degradation of NS2B/3 to its antiviral restriction capacity in the context of flaviviruses.

In the context of retroviruses, capsid binding by cyclophilin A (CypA) is required for virus replication (Gamble et al., 1996; Luban et al., 1993), and substitution of the B30.2/SPRY domain of hTRIM5α with CypA facilitates hTRIM5α binding to HIV-1 and virus restriction (Luban, 2007, 2012). The tick-borne flaviviruses, including LGTV, are sensitive to Cyp inhibition (Figure S5A; Chiramel et al., 2016), and CypA specifically is required for efficient virus replication (Figure S5B). However, although substitution of owl monkey CypA (Sayah et al., 2004) or human CypA (Gamble et al., 1996) for the hTRIM5α B30.2/SPRY domain suppressed replication of VSV-G pseudotyped HIV-1 (Figures S1A and S1B), these fusion proteins had no effect on replication of LGTV (Figures S5C and S5D). Thus, although CypA is required for flavivirus replication and binds to NS proteins NS5 (Qing et al., 2009) and NS4B (Vidotto et al., 2017) within viral replication complexes, TRIM5-CypA fusion proteins are not sufficient to restrict tick-borne flavivirus replication, confirming the importance of the B30.2/SPRY domain of TRIM5α in flavivirus restriction.

### Endogenous hTRIM5α Is an Antiviral Restriction Factor for Flaviviruses

**Figure 7.**
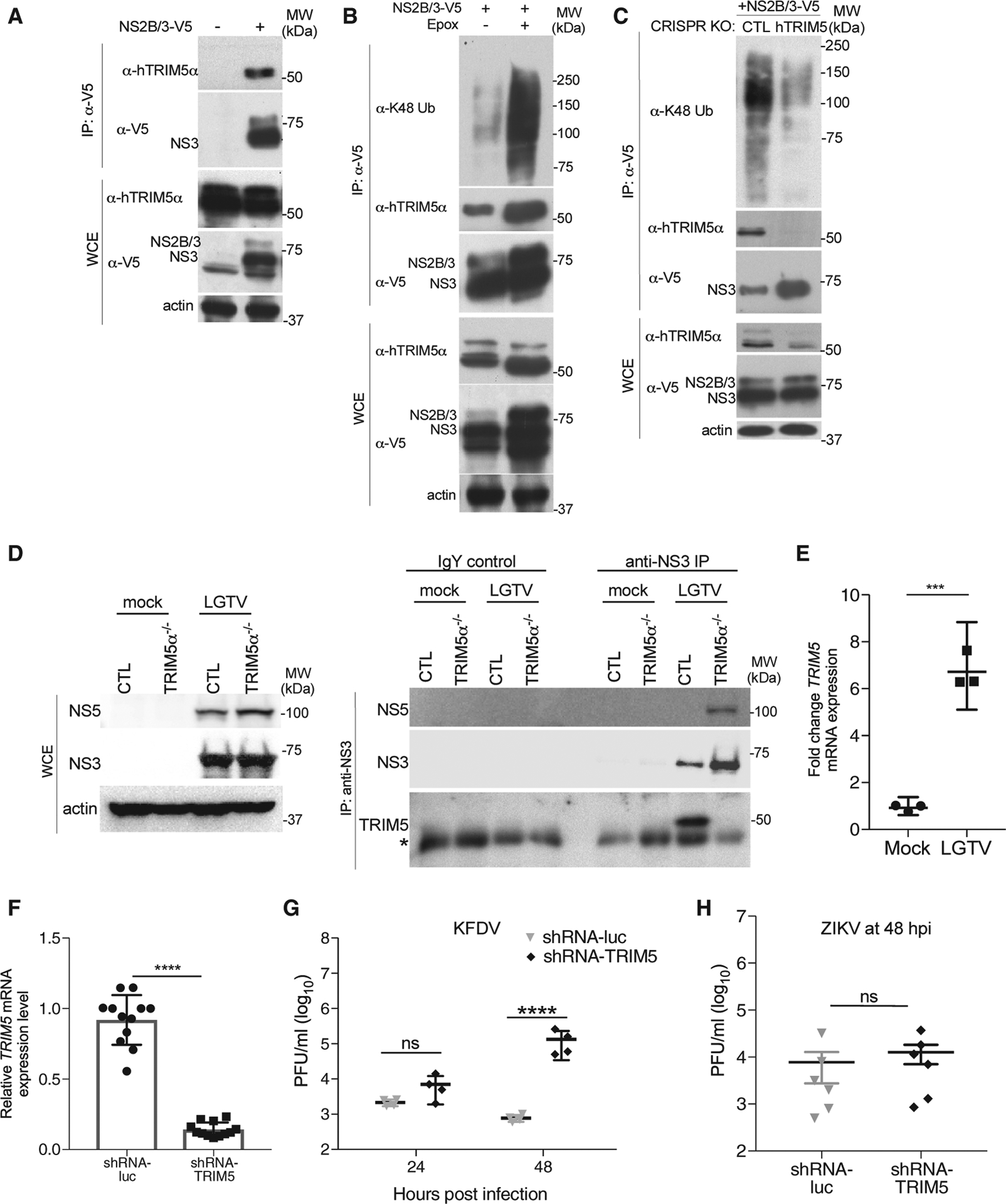
Endogenous hTRIM5α Is an Antiviral Restriction Factor for Flaviviruses (A) IP of LGTV NS2B/3-V5 following ectopic expression in unmodified HEK293 cells and probed for TRIM5. (B) IP of LGTV NS2B/3-V5 following ectopic expression and epoxomicin treatment in unmodified HEK293 cells. Western blots were probed for TRIM5 and K48-linked ubiquitin. (C) IP of LGTV NS2B/3-V5 following ectopic expression in HEK293 cells transfected with plasmids encoding TRIM5 gRNA and Cas9. Western blots were probed for TRIM5 and K48-linked ubiquitin. (D) HAP1 control and TRIM5−/− cells were infected with LGTV (MOI 0.1), and NS3 was immunoprecipitated at 48 hpi. Western blots were probed for TRIM5, NS3, and NS5. (E and F) TRIM5 mRNA expression in primary human MDDCs (E) infected with LGTV (MOI 5 at 24 hpi) or (F) transduced with lentiviruses expressing shRNA-Luc (control) or shRNA-TRIM5 (mean ± SD from 3 experiments in triplicates, *p < 0.0001, unpaired t test). (G) KFDV titers or (H) ZIKV titers following infection of human MDDCs generated in (F). (MOI of 0.1; mean ± SD from 1 of 2 experiments performed, ****p < 0.0001 one-way ANOVA with Sidak post-test).

The role of hTRIM5α in suppression of HIV-1 has been controversial, in part because early studies suggested no restriction of laboratory strains of HIV-1. However, recent studies suggest that cytotoxic T lymphocyte (CTL)-selected HIV-1 isolates from so-called “elite controllers” are susceptible to restriction by hTRIM5α (Merindol et al., 2018), and genetic studies suggest that human polymorphisms in *TRIM5* impact disease progression (Merindol and Berthoux, 2015). To further examine whether TRIM5α in human cells restricts flavivirus replication, we first immunoprecipitated LGTV NS2B/3 following ectopic expression in unmodified HEK293 cells, which revealed an interaction with endogenous TRIM5α (Figure 7A). Treatment of these cells with epoxomicin increased the levels of co-precipitating TRIM5 and NS2B/3 as well as the presence of endogenous K48-linked Ub smears in the complex (Figure 7B), whereas depletion of TRIM5α by CRISPR/Cas9-mediated gene editing both increased levels of NS3 and decreased endogenous K48-linked Ub smears in the precipitates (Figure 7C). Endogenous interactions between NS3 and TRIM5α were also confirmed in the HAP1 cells knocked out for TRIM5α by CRISPR/Cas9 and infected with LGTV (Figure 7D). Finally, infection of primary human-monocyte-derived dendritic cells (DCs) resulted in upregulation of *TRIM5* expression (Figure 7E). Silencing of *TRIM5* expression in human DCs by lentivirus-delivered shRNA expression (Pertel et al., 2011) increased the release of infectious KFDV by approximately 170-fold at 48 hpi compared to cells expressing shRNA specific for luciferase as a control (Figures 7F and 7G). No effect of TRIM5α silencing was observed following infection with ZIKV (Figure 7H). Together, these data demonstrate that hTRIM5α is a bona fide restriction factor for specific flaviviruses that functions through interactions with the viral replication complex and proteasomal degradation of NS3.

## DISCUSSION

TRIM5α functions as an intrinsic cellular restriction factor that recognizes retrovirus capsids with high specificity and with definitive consequences for primate susceptibility to HIV-1 infection (Ganser-Pornillos et al., 2011; Hatziioannou et al., 2006; Merindol and Berthoux, 2015; Stremlau, 2007; Stremlau et al., 2004; Stremlau et al., 2006; Wu et al., 2013). Here, we show that both hTRIM5α and rhTRIM5α restrict replication of specific flaviviruses within the TBEV serocomplex and that endogenous TRIM5α is required for the antiviral effects of type I IFN against sensitive flaviviruses in human cells. The viruses sensitive to TRIM5α included TBEV, KFDV, and LGTV but interestingly not POWV. However, mosquito-borne YFV, DENV, ZIKV, and WNV were not sensitive to rhTRIM5α-mediated restriction. We further identified the viral protease NS2B/3 as a major target recognized by the SPRY/B30.2 domain of TRIM5α. NS2B/3 is responsible for a number of cleavage events of the viral polyprotein, and NS3 additionally encodes the RNA helicase essential to viral RNA replication (Lindenbach et al., 2007). NS3 also contributes to virus particle assembly (Gebhard et al., 2016), and NS2B/3 has been shown for some flaviviruses to cleave host proteins involved in antiviral sensing and production of type I IFN (Aguirre et al., 2012; Yu et al., 2012). Thus, the essential role of NS2B/3 in virus replication explains the generalized effect of TRIM5α in reducing viral RNA replication, protein expression, and production of infectious progeny. Taken together, this work significantly extends the paradigm of TRIM5α as an antiviral restriction factor and suggests that, in contrast to the current view, TRIM5α exhibits a remarkable plasticity in recognition of unrelated viruses.

The precise molecular determinant recognized by TRIM5α was not finely mapped but required both the cytoplasmic domain of NS2B that contributes to NS3 protease structure and function, as well as the linker region between protease and helicase domains. The linker domain is functionally important in directing the conformation of the viral helicase relative to the ER membrane and to the protease domain in order to regulate polyprotein processing and genome replication (Luo et al., 2010). Thus, binding of the linker domain by TRIM5α may result in steric hindrance of NS3 function in addition to protein degradation. Indeed, mutation of the TRIM5α RING domain prevented NS3 degradation but was still able to impact a weak level of restriction of LGTV replication. The requirement for NS2B sequences that contribute to the protease structure suggests that the binding requirements of TRIM5α are dependent on NS2B/3 conformation. Interestingly, targeting the E3 ligase activity of TRIM5 to sites of tick-borne flavivirus replication through fusion with CypA is not sufficient to restrict infection, despite a clear requirement for CypA in flavivirus replication (Chiramel et al., 2016; Qing et al., 2009; Vidotto et al., 2017), perhaps because CypA binds to different NS proteins (NS5, NS4A) (Qing et al., 2009; Vidotto et al., 2017) than does TRIM5α. These results suggest highly coordinated activities of the SPRY/B30.2 and RING domains in NS2B/3 binding and effector functions, respectively. Therefore, thorough mapping and structural studies are required to precisely delineate TRIM5α interactions with NS2B/3 and how this compares with known structural determinants governing the TRIM5α interaction with retrovirus capsid lattices.

The rapid evolution of the *TRIM5* gene throughout primate evolution is associated with selection pressure from lentivirus capsid sequences (McCarthy et al., 2015). It is, therefore, unclear how evolutionary selection of TRIM5α for retrovirus restriction has left the protein with enough flexibility to maintain antiviral activity against flaviviruses. It may be possible that ancient flavivirus-like viruses have influenced the evolution of hTRIM5α. However, the time frame of flavivirus evolution is in the order of thousands of years in contrast to millions of years for retroviruses and the TRIM5 gene (Kaiser et al., 2007). *Flaviviridae* includes the more ancient genera of Hepaciviruses, although evidence for a zoonotic origin of hepatitis C virus (HCV) in non-human primates is not strong despite the extremely narrow host range of HCV limited to humans and chimpanzees (Simmonds, 2013). Therefore, it seems unlikely that flaviviruses influenced the positive selection of the *TRIM5* gene within the human lineage. However, our work raises the possibility that human polymorphisms within the *TRIM5* locus could influence resistance to infection with medically important flaviviruses. Thus, understanding the genetic trade-offs in both TRIM5α and NS2B/3 that enable restriction of flaviviruses versus retroviruses represent an important model to illustrate how host resistance is shaped by multiple pathogens and might provide new insight to human susceptibility to emerging flaviviruses.

Among the tick-borne flaviviruses tested, POWV was insensitive to TRIM5α-mediated restriction. This raises the question of how flaviviruses escape restriction and whether this has implications for host tropism and evolution of these viruses. Despite the close genetic relationship between POWV and other tick-borne viruses sharing ~80%–87% amino acid identity across NS3, it is possible that encodes NS2B/3 sequences that are not bound by TRIM5α, as was observed for WNV. Alternatively, virus-specific interactions between the flavivirus replication complex and CypA might protect against TRIM5 binding, as was recently reported for HIV-1 (Kim et al, 2019). Finally, it is possible that some flaviviruses may encode an antagonist of TRIM5α activity. Detailed mapping studies to identify viral determinants of TRIM5α binding and degradation will shed light on these potential evasion strategies and will further enable studies examining the role of TRIM5α in primate immunity to infection.

Although primates have a single TRIM5 gene, rodents have an expanded clade of TRIM5 genes with at least seven TRIM5 paralogs in mice that cluster into two groups designated TRIM30 and TRIM12 (Tareen et al., 2009). TRIM12 genes possess anti-retroviral activity, although a function in retrovirus restriction has not been observed for TRIM30 genes (TRIM30-a, -b, -c, and -d) (Lascano et al., 2015; Tareen et al., 2009). We previously demonstrated that TRIM30-d (also known as TRIM30-3 or TRIM79) restricts replication of a subset of flaviviruses within the TBEV serogroup (Taylor et al., 2011). TRIM30-d interacted with and degraded the viral NS5 protein that is essential for virus replication owing to its MTase and RdRP functions and its role as an antagonist of IFN-I-dependent signaling (Best, 2017; Best et al., 2005; Laurent-Rolle et al., 2010). TRIM30-d directed the destruction of NS5 complexed with NS3 by lysosomes, consistent with the ability of lysosomes to accommodate the degradation of large protein complexes (potentially through autophagy) (Mandell et al., 2014b; Sparrer et al., 2017), although TRIM30-d did not degrade NS2B/3 expressed in the absence of NS5 (Taylor et al., 2011). Like TRIM5α, restriction of virus replication by TRIM30-d appeared specific to tick-borne flaviviruses, including LGTV, POWV, and TBEV, because TRIM30-d did not inhibit the replication of WNV (Taylor et al., 2011). Rodents are required for the maintenance of tick-borne flaviviruses in nature by enabling tick co-feeding and transfer of virus from infected to uninfected ticks in the absence of detectable viremia (Kazimírová et al., 2017). However, despite the parallels in antiviral activity between TRIM30-d and TRIM5α, it is unlikely that flavivirus sensitivity to TRIM30-d directly influenced sensitivity to primate TRIM5α, as the targeted viral proteins and mechanisms of antiviral activity are unrelated. Instead, the evolutionary history of tick-borne flaviviruses in rodents suggests that the TRIM30-d/NS5 interaction has arisen independently to TRIM5/NS2B-3 interactions.

In summary, the finding that primate TRIM5α can recognize and degrade NS2B/3 from specific flaviviruses combined with a strong antiviral role in the type I IFN response suggests that TRIM5 has a high potential to function as an important human barrier to infection with emerging flaviviruses. We speculate that resistance to TRIM5α-mediated restriction may be an important factor in enabling the use of primates as reservoirs for viruses such as YFV, DENV, and ZIKV. Regardless, these findings reveal an alternative model to retroviruses to explore the structure and function of TRIM5α in human resistance to virus infection.

## STAR★METHODS

### CONTACT FOR REAGENT AND RESOURCE SHARING

Further information and requests for resources and reagents should be directed to and will be fulfilled by the Lead Contact, Sonja Best (sbest@niaid.nih.gov).

### EXPERIMENTAL MODEL AND SUBJECT DETAILS

#### Cell Culture

HEK293T cells (human embryonic kidney, ATCC; CRL-3216 - fetus sex unknown), HEK293 cells (human embryonic kidney, ATCC; CRL-1573 - fetus sex unknown), CRFK cells (feline kidney - female, ATCC; CCL-94), A549 cells (lung carcinoma - male, ATCC; CCL-185) and Vero (sex unknown – ATCC; CCL-81) cells were cultured in Dulbecco’s modified Eagle media (GIBCO; 11995) supplemented with 10% fetal bovine serum (GIBCO; 16000-044), 2 mM L-glutamine (Invitrogen; 25030-081), and 1% antibiotics (GIBCO; 15140) (complete media) at 37°C and 5% CO_2_. Near-haploid human cell line derived from male chronic myelogenous leukemia (CML) - HAP1 cells (Horizon Discovery) was cultured in complete IMDM (GIBCO; 12440053) supplemented with 10% fetal bovine serum, 2 mM L-glutamine and 1% antibiotics. Human monocyte derived dendritic cells were from both male and female donors.

#### Generation of human monocyte derived dendritic cells (DCs)

Human monocytes enriched by apheresis were obtained from peripheral blood provided by the Department for Transfusion Medicine and the National Institutes of Health Clinical Center (NIHCC) at the National Institutes of Health (NIH) [Bethesda, MD] under a protocol approved by the NIHCC Institutional Review Board. Signed, informed consent was obtained from each donor, acknowledging that his or her donation would be used for research purposes by intramural investigators throughout the NIH. Monocytes were further enriched using Ficoll-Paque PREMIUM (GE Healthcare) and were differentiated into hDCs following culture in RPMI 1640 supplemented with 10% heat-inactivated FCS, 0.2 mM l-glutamine, 1 mM HEPES buffer, and 0.1 mM nonessential amino acids [complete RPMI 1640 (cRPMI)] and 100 ng/ml GM-CSF (R&D Systems, #215-GM) and 20 ng/ml IL-4 (R&D Systems, #204-IL) over the course of 4 d. On day 3 of culture, 100% of each cytokine per well in 1 mL cRPMI was added and cells were used on day 4 of culture. The resulting differentiated hDCs were > 97% CD1a^+^/DC-SIGN^+^ and < 1% CD14^+^. The hDCs were seeded at 5 × 10^5^cells per milliliter in cRPMI.

### METHOD DETAILS

#### Inhibitors

Cell culture grade proteasomal inhibitors epoxomicin (#E3652) and MG132 (#CAS 133407-82-6) were used at 200nM (4 hours) and 10 μg (4 hours) respectively. Lysosomal inhibitor bafilomycin A1 (Baf-A1) (#B1793) was used at 200nM (4 hours).

#### Virus Infections and Lentivirus production

The viruses used in this study were handled under biosafety level 2 (BSL2), BSL3 and BSL4 conditions at the Rocky Mountain Laboratories Integrated Research Facility in accordance with DSAT regulations for study of select agents and Institutional Biosafety approvals (Hamilton, MT). The viruses in this study include: Langat virus (LGTV) strain TP21 (from Dr. A. Pletnev, NIAID, NIH), TBEV strain Sofjin (also referred to as Russian spring summer encephalitis [RSSE] virus), Kyasanur forest disease virus (KFDV) [from Dr. M. Holbrook, NIAID, NIH], Powassan virus (POWV, strain LB) and West Nile virus (strain NY99) [from the WRCEVA], Dengue virus (DENV-2, strain New Guinea C) from Dr. Adolfo García-Sastre), Zika virus (ZIKV, strain 2013 French Polynesia, from Dr. David Safronetz) and Yellow fever virus (YFV, strain 17D), from NIH Biodefense and Emerging Infections Research Resources Repository, NIAID, NIH, NR115. All viruses were propagated as previously described (Taylor et al., 2011). Cell monolayers were infected for 1 h at 37°C, after which virus inoculum was removed and cells replenished with fresh cell culture medium. Virus titers are represented as plaque forming units (PFUs) or focus forming units (FFU) per 1 ml.

HIV-1 virus pseudotyped with VSV-G and encoding a GFP reporter for single-cycle infection assays were packaged in 293T cells seeded at a concentration of 1×10^6^ cells/well in a 6-well dish. One day after seeding, cells were co-transfected with 0.5 μg pMDLg/pRRE, 0.25μg pRSV-Rev, 0.2μg pMD2.G, and 1μg pRRLSIN.cPPT.PGK-GFP.WPRE (plasmids 60488, 12253, 12252 respectively available from Addgene). Cells were transfected using TransIT-293 at a 1:3 ratio (μg DNA:μl TransIT-293). After 48 hours, supernatant containing viruses was harvested, filtered, and frozen. For infection assays, CrFK stable cells lines were plated at a concentration of 7.5×10^4^ cells/well in a 24-well plate or HEK293 stable cell lines were plated at a concentration of 1.0×10^5^ cells/well in a 24-well plate, and infected with HIV-1 single-cycle virus. Two days post-infection, cells were fixed, washed, resuspended in PBS supplemented with 1% FBS, and analyzed by flow cytometry for expression of GFP using the BD Bioscience Fortessa cell analyzer.

##### Lentivirus generation expressing shRNAs

The shTRIM5 and shluciferase lentiviruses were generated by transfecting HEK293T cells with lentivirus shRNA plasmid (pAPM CoE D4 L1221 or pAPM CoE D4 TRIM5 ts2 for shluciferase or shTRIM5, respectively), pSPAX2, and pMD.G using the ProFection Mammalian Transfection System (Promega). pAPM CoE D4 is a truncated derivative of the pAPM lentiviral vector that expresses the puromycin acetyltransferase and miR30-based shRNA from the SFFV promoter (Pertel et al., 2011). The target sequences are: pAPM CoE D4 L1221 5′-TACAAACGCTCTCATCGACAAG-3′ and pAPM CoE D4 TRIM5 ts2 5′-TGCCAAGCATGCCTCACTGCAA-3′. The vpx-vlp was generated by transfecting 293T cells with pMD.G and SIV_MAC_ packaging plasmid kindly provided by Dr. Andrea Cimarelli (Berger et al., 2011). Media was replaced 18–20 hours post transfection (hpt). Supernatant was harvested at 48 hpt, passed through a 0.45 um filter, and ultracentrifuged over a cushion consisting of 25% sucrose in TNE buffer (10 mM Tris-HCl, pH 7.5, 1 mM EDTA, 100 mM NaCl, pH 7.4) at 28,000 rpm in a SW-28 Rotor (Beckman). Lentivirus pellets were resuspended in PBS, aliquoted, and stored at −80°C prior to use. shRNA-luc and shRNA-TRIM5 lentivirus titers were normalized by serial dilution on HEK293 cells followed by puromycin selection.

##### Knockdown of TRIM5 in Human monocyte-derived dendritic cells (hMDDC) cultures

Human monocyte cultures (Ireland et al., 2018) were seeded in 48-well plates and transduced with a combination of vpx-vlp and shControl or shTRIM5 lentivirus for three hours followed by addition of IL-4 and GM-CSF-conditioned RPMI media. Conditioned media was replenished at 3 days post transduction (dpt). Five dpt, cells were collected to confirm knockdown of TRIM5 transcripts by qRT-PCR. Remaining cells were infected with ZIKV PRABC59 (MOI = 5) or KFDV (MOI = 0.1) for 48 hours. Supernatants were collected at the indicated times, and virus was measured in the supernatant by limiting dilution plaque assay.

#### Expression constructs

HA-tagged (C-term) human and rhesus *TRIM5* in the pLPCX retroviral vector were obtained from the National Institutes of Health AIDS Research and Reference Reagent Program. HA-tagged (C-term) owl monkey *TRIM-CypA* in the pLPCX retroviral vector was a kind gift from Dr. Michael Emerman (Fred Hutchinson Cancer Research Center). Approximately 5×10^6^ HEK293 cells were used to isolate RNA with the All Prep RNA/DNA Mini Kit (QIAGEN; 80204). cDNA was generated using 1 μg of RNA with oligo(dT) primers and the Superscript III First-Strand Synthesis System (Invitrogen; 18080-051). This cDNA was used as a template to amplify the *CypA* coding region (see below). All primers used in this study for to generate constructs or qRT-PCR, along with a description of their use, can be found in Tables S1 and S3. Human *TRIM22* was amplified from a pcDNA3 construct kindly provided by Dianne Lou. *TRIM-CypA* constructs were generated by amplifying fragments (aa 1-309 from human *TRIM5* in pLPCX and the complete coding sequence of *CypA* from HEK293 cDNA) with 20–25bp overlapping regions. Overlapping fragments were spliced together in a PCR reaction using each fragment as a template and outside flanking primers. Human and rhesus *TRIM5delB30.2* constructs were generated using pLPCX templates and primers that amplify aa 1-276 from human *TRIM5* or 1-278 from rhesus *TRIM5*. All above PCR reactions were carried out using PCR Supermix High Fidelity (Thermo Fisher; 10790020) with an annealing temperature of 58°C. Constructs were TA-cloned into the gateway entry plasmid pCR8 (Invitrogen; K2500-20). An LR Clonase II reaction (Invitrogen; 11791-100) was used to move these constructs into a Gateway-converted pLPCX retroviral packaging vector (Clontech; 631511). The RING C15/18A mutant of *TRIM5* was generated using PfuTurbo DNA polymerase (Stratagene; 600250) with an annealing temperature of 55°C. Parental pLPCX plasmids were used as a template along with primers containing the mutations of interest. Constructs expressing LGTV and WNV_NY99_ NS2B/3 and NS5 were generated as previously described (Taylor et al., 2011). Expression plasmids for Langerin (HG13040-UT) and DC-SIGN (HG10200-UT) were purchased from Sino Biological.

#### Generation of stable cells lines

To make cell lines that stably express *TRIM5* constructs, pLPCX retroviral vectors were used to transduce HEK293 cells. To generate the retroviruses used for transduction, HEK293T cells were seeded at a concentration of 1×10^6^ cells/well in a 6-well dish. 24 hours later each well was transfected with 2 μg pLPCX construct (empty or encoding the gene fragment of interest), 1 μg pCS2-mGP encoding MLV gag-pol^2^, and 0.2 μg pC-VSV-G (provided by Hyeryun Choe) at a final 1:3 ratio of DNA to TransIT-293 (μg DNA: μl TransIT-293). Supernatants were collected after 48 h, passed through a 0.2 μm filter, and used to infect HEK293 cells grown in complete media. HEK293 cells were seeded in a 12-well dish at a concentration of 7.5×10^4^ cells/well. After 24 h, varying amounts of retrovirus from each construct were added to cells along with polybrene (Sigma; 107689) at a final concentration of 10 μg/mL. After 24 h, media containing 0.75 μg/ml puromycin (Sigma; P8833) was added to select for transduced cells. Cell lines were eventually expanded into 10 cm dishes, checked for expression of the appropriate construct by western blot, and frozen down in 1 mL aliquots containing complete media supplemented with an additional 10% FBS (total of 20%) and 5% DMSO. A549 cells were stably knocked-down using lentiviruses coding short hairpin RNAs (shRNAs) against *Cyclophilin A*, *B* and *non-targeting* (control) *as* previously described (kindly provided by Prof. Ralf Bartenschlager) (Kaul et al., 2009). HAP1 cells edited within the TRIM5 gene were generated by Horizon Genomics (Vienna) with the RNA guide sequence: CGATTAGGCCGTATGTTCTC.

#### Antibodies

HA-tagged constructs for western blotting were detected using a 1:5000 dilution of anti-HA-peroxidase antibody (Roche clone 3F10, #12013819001). HA-tagged constructs for indirect immunofluorescence were detected using anti-HA (Zymed, #71-5500). β-actin was also detected as a loading control using a 1:10,000 dilution of mouse anti-β-actin (Sigma, A5441). A 1:3,000 dilution of goat anti-mouse (Dako, #P0447), anti-rabbit (Thermo Scientific, #P0448) or anti-chicken (Millipore, #12-341) horseradish peroxidase-conjugated antibody was used as a secondary probe. V5 tagged constructs were probed with anti-mouse V5 (Invitrogen #R960-25). Blots were developed using the ECL Plus detection reagent (GE Healthcare, #RPN2132). Antibodies to detect viral antigens, LGTV (NS3 and NS5) (previously described in Taylor et al., 2011), WNV-NS3 (R&D Systems, #AF2907) and dsRNA antibody J2 (English& Scientific Consulting, #10010200). Autophagy and cellular markers were detected using LC3B (Nanotools, #5F10), GABARAP (Cell Signaling, #E1J4E), Beclin-1 (Novus Biologicals, # 110-53818), ATG5 (Cell Signaling, #2630), p62 (BD Transduction Laboratories, #610833), cyclophilin A (Enzo, #BML-SA296-0100), cyclophilin B (Thermo Scientific, #PA1-027A), langerin (R&D Systems, #AF2088) and DC-SIGN (BD Biosciences, #551186).

#### Immunoprecipitation (IP) and Western Blot Analysis

293 cells were washed three times with PBS (1X) and lysed on ice in RIPA buffer (50 mM Tris-HCl [pH 7.6], 150 mM NaCl, 0.1% SDS, 1% Igepal, and 0.5% Na-deoxycholate) with protease inhibitor cocktail (Roche). For IPs of overexpressed proteins, 2 wells of a 6 well dish at 1×10^6^ cells/well were used per reaction; for IPs of virus-infected stable TRIM5 HEK293 cells, a 10cm dish of 7×10^6^ cells/dish was used per reaction; for detection of endogenous TRIM5, HEK293 or HAP1 cells were grown to confluency in 3–4 T150 tissue culture flasks. Samples were subjected to centrifugation for 10 min at maximum speed to remove cellular debris. Protein G-conjugated agarose beads (Roche) or PrecipHen for chicken antibodies (Aves Labs) were used to clear cell lysates at 4°C for 3 h. Samples were centrifuged to remove beads, and 2 μg of antibody analogous to the protein of interest was added to each lysate for 1 h with rotation at 4°C. 50 μL protein G-agarose or PrecipHen beads and were incubated with rotation at 4°C overnight. Lysates were subjected to centrifugation, and beads were washed three times with RIPA buffer prior to elution by incubation at 95°C in 1 3 sample buffer (62.5 mM TRIS [pH 6.8], 10% glycerol, 15 mM EDTA, 4% 2-ME, 2% SDS, and bromophenol blue). For western blot analysis HEK293 cell lines were grown to confluency in a 12-well or 6-well dish, collected using a cell scraper, and lysed in RIPA buffer containing complete protease inhibitor (Roche, #11836170001). After quantification of protein concentration using a Bradford assay, 30 μg of whole cell extract was resolved using a 10% polyacrylamide gel and transferred to a nitrocellulose membrane. Ubiquitination assays were performed as previously described (Campbell et al., 2015). Densitometry analysis was performed using ImageJ software.

#### Confocal Microscopy

Cells were seeded onto 4 well Lab-Tek II chamber slides overnight. Slides were prepared by washing cells twice with PBS (1X) and subsequently fixed with paraformaldehyde (4%) for 10 min. For double-stranded RNA (dsRNA) staining, cells were fixed with methanol (100%) for 5 min at −20°C. Slides fixed with paraformaldehyde (4%) were further incubated with permeabilization buffer (Triton X-100 [0.1%], sodium citrate [0.1%]) for 5 min at room temperature and incubated with blocking buffer (PBS[1X], BSA [0.5%] and goat serum [1%]) for 30 min. Cells were incubated with primary antibody overnight at 4°C, washed three times with PBS (1X) and further incubated with secondary antibody conjugated to Alexa 488, - 594 or −647 (Molecular Probes) for 1 h. Slides were washed three time with PBS(1X) and once with ddH_2_0, and mounted onto glass coverslips using Prolong Gold + DAP1 mounting media (Molecular Probes). Processed slides were imaged using a Zeiss LSM710 confocal microscope and vector profiles analyzed using Zen software (Carl Zeiss).

#### Flow cytometry

Cells were harvested at 48 hpi and processed for flow cytometry analysis. Cells were stained with LIVE/DEAD Fixable Aqua Dead Cell Stain Kit (ThermoFisher) and fixed with 4% paraformaldehyde for 20 min at RT. Cells were permeabilized with saponin-containing buffer and probed with anti-E 11H12 antibody. Data were generated using an LSRII flow cytometer (BD Biosciences) and analyzed using FlowJo (Tree Star).

#### RNA Isolation and quantitative RT-PCR

RNA was isolated from cells using RNeasy kit (QIAGEN) and genomic DNA was removed with RNase-free DNase (QIAGEN). Reverse transcription of RNA was performed using Superscript Vilo cDNA Synthesis Kit (Invitrogen) according to manufacturer’s protocol. TaqMan probes (Table S1) specific for *TRIM5*, hypoxanthine-guanine phosphoribosyltransferase (*HPRT*), interferon beta (*IFNβ*), interlukin −6 (*IL6*), tumor necrosis factor alpha (*TNFα*) and C-X-C motif chemokine 10 (*CXCL10*) were obtained from Applied Biosystems. Reactions for Real-time RT-PCR were set up in triplicate, cycled and data was collected on the Applied Biosystems GeneAmp 9500 Sequence detection system. Quantification of relative gene expression was relative to untreated controls with comparative C_T_ method.

#### RNA interference

HEK293 and A549 cells were transfected with 15 pmol of siRNA using Lipofectamine RNAiMAX (Life Technologies), refer Table S2. siRNAs (Dharmacon; SMART pool) were specific against TRIM5 (L-007100), LC3B (L-012846), GABARAP (L-012368), Beclin-1 (L-010552), ATG5 (L-004374) and p62 (L-010230).

#### Quantification and statistical analysis

All data were evaluated for significance using one-tailed unpaired Student’s t test, or Mann-Whitney U test or one-way/two-way ANOVA GraphPad Prism 7 software. The number of experimental and technical replicated for statistical analysis is indicated in each figure legend. Data involving virus titrations were generated with values over 2–3 experimental replicates performed in triplicates and analyzed for significance using Mann-Whitney U test or one-way ANOVA post-test as indicated, in figure legends. Western blots and experiments involving immunofluorescence were generally performed a minimum of two times, unless quantification was performed in which case the number of experiments is listed in the figure legends. Quantitative image analysis were measured along vectors drawn in at least 3 fields of cells and validated for significance using two-way ANOVA with Sidak’s or Dunnett’s post-test, as indicated in figure legends.

## Supplementary Material

1

## Figures and Tables

**Table:** KEY RESOURCES TABLE

REAGENT or RESOURCE	SOURCE	IDENTIFIER
Antibodies		
Anti-HA-peroxidase (Clone 3F10)	Roche	Cat#12013819001; RRID: AB_390917
Anti-HA	Zymed	Cat #71-5500; RRID: AB_87935
Anti-β-actin	Sigma	Cat #A5441; RRID: AB_476744
Goat anti-mouse	Dako	Cat #P0447; RRID: AB_2617137
Anti-rabbit	Thermo Scientific	Cat #P0448; RRID: AB_2617138
Anti-chicken horseradish peroxidase-conjugated	Millipore	Cat #12-341; RRID: AB_390189
Anti-mouse V5	Invitrogen	Cat #R960-25; RRID: AB_2556564
Anti-chicken LGTV NS3	Customized (Dr. Sonja Best)	Previously described in Taylor et al., 2011
Anti-chicken LGTV NS3	Customized (Dr. Sonja Best)	Previously described in Taylor et al., 2011
Anti-NS3-WNV	R&D Systems	Cat #AF2907; RRID: AB_562749
Anti-dsRNA antibody J2	English& Scientific Consulting	Cat #10010200; RRID: AB_2651015
Anti-LC3B	Nanotools	Cat #5F10; RRID: AB_2722733
Anti-GABARAP	Cell Signaling	Cat #E1J4E; RRID: AB_2798306
Anti-Beclin-1	Novus Biologicals	Cat # 110-53818; RRID: AB_1726526
Anti-ATG5	Cell Signaling	Cat #2630; RRID: AB_2062340
Anti-p62	BD Transduction Laboratories	Cat #610833; RRID: AB_398152
Anti-cyclophilin A	Enzo Life Sciences	Cat #BML-SA296-0100; RRID: AB_2051206
Anti-cyclophilin B	Thermo Scientific	Cat #PA1-027A; RRID: AB_2169138
Anti-langerin	R&D Systems	Cat #AF2088; RRID: AB_355143
Anti-DC-SIGN	BD Biosciences	Cat #551186; RRID: AB_394087
Secondary Antibody Alexa 488 (Rabbit)	Molecular Probes	Cat #A11034; RRID: AB_2576217
Secondary Antibody Alexa –568 (Mouse)	Molecular Probes	Cat #A11031; RRID: AB_144696
Secondary Antibody Alexa-647 (Chicken)	Molecular Probes	Cat #A21449; RRID: AB_1500594
Virus Strains		
Langat virus (LGTV)	Dr. A. Pletnev, NIAID, NIH	strain TP21
TBEV (also referred to as Russian spring summer encephalitis [RSSE] virus)	Dr. M. Holbrook, NIAID, NIH	strain Sofjin
Kyasanur forest disease virus (KFDV)	Dr. M. Holbrook, NIAID, NIH	Strain P96056
Powassan virus (POWV)	WRCEVA, UTMB	strain LB
West Nile virus	WRCEVA, UTMB	strain NY99
Dengue virus	Dr. Adolfo Garcıá-Sastre	DENV-2, strain New Guinea C
Zika virus (ZIKV)	Dr. David Safronetz	strains 2013 French Polynesia and PRABC59
Yellow fever virus (YFV)	NIH Biodefense and Emerging Infections Research Resources Repository, NIAID, NIH, NR115	strain 17D
HIV-1 virus pseudotyped with VSV-G	Dr. Nicholas Meyerson and Dr. Sara Sawyer	Universoty of Colorado, Boulder, pseudotyped with VSV-G and encoding a GFP reporter
Chemicals, Peptides, and Recombinant Proteins		
2 mM L-glutamine	Invitrogen	Cat #25030-081
Epoxomicin	Sigma	Cat #E3652
Dulbecco’s modified Eagle media	GIBCO	Cat #11995
MG132	Sigma-Aldrich (Calbiochem)	Cat #CAS 133407-82-6
Bafilomycin A1 From Streptomyces Griseus	Sigma-Aldrich	Cat #B1793
Penicillin	GIBCO	Cat #15140
Puromycin	Sigma	Cat #B9620
Blasticidin	Sigma	#CAS 2079-00-7
Polybrene	Sigma	Cat #107689
Protease inhibitor	Roche	Cat #11836170001
Protein G-conjugated agarose beads	Roche	Cat #11719416001
Preciphen®	Aves, Lab Inc.	Cat # P-1010
RIPA buffer	Sigma	Cat #R0278-500
ProLong Gold Antifade Mountant with DAPI	ThermoFisher Scientific	Cat #P36931
(Granulocyte-Macrophage Colony Stimulating Factor) GM-CSF	R&D Systems	Cat #215-GM
(Interleukin-4) IL-4	R&D Systems	Cat #204-IL
RNase-free DNase	ThermoFisher Scientific	Cat #EN0521
Critical Commercial Assays		
Live/Dead Fixable Aqua Dead Cell Stain Kit	ThermoFisher Scientific	Cat #L34957
Interferon β (IFNβ)	PBL Assay Science	Cat #11410-2
ECL Plus detection reagent	GE Healthcare	Cat #RPN2132
Lipofectamine RNAiMAX Transfection Reagent	Thermo Fisher Scientific	Cat #13778075
Prep RNA/DNA Mini Kit	QIAGEN	Cat #80204
Superscript III First-Strand Synthesis System	Invitrogen	Cat #18080-051
ProFection Mammalian Transfection System	Promega	Cat #E1200
PCR Supermix High Fidelity	Thermo Fisher	Cat #10790020
Clonase II reaction	Invitrogen	Cat #11791-100
PfuTurbo DNA polymerase	Stratagene	Cat #600250
RNeasy kit	QIAGEN	Cat #74104
PlasmoTest-Mycoplasma Detection	Invivo Gen	Cat #rep-pt1
Experimental Models: Cell Lines		
HEK293T	ATCC	CRL-3216
HEK293	ATCC	CRL-1573
CRFK cells (feline kidney)	ATCC	CCL-94
A549	ATCC	CCL-185
Vero	ATCC	CCL-81
Oligonucleotides		
Probes for qRT-PCR used in this study	Applied Biosystems	Table S1
siRNAs used in this study (SMARTpool)	Horizon (Dharmacon)	Table S2
Primers for cloning TRIM5 constructs	Integrated DNA Technologies	Table S3
shRNA sequence of lentiviral constructs (Control – Luciferase)	This paper	TACAAACGCTCTCATCGACAAG
shRNA sequence of lentiviral constructs (human TRIM5)	This paper	TGCCAAGCATGCCTCACTGCAA
Recombinant DNA		
pLPCX Empty Vector	Clontech	Cat #519 631511
pLPCX human TRIM5	National Institutes of Health AIDS Research and Reference Reagent Program.	N/A
pLPCX rhesus TRIM5	National Institutes of Health AIDS Research and Reference Reagent Program.	N/A
pLPCX owl monkey TRIM-CypA	Dr. Michael Emerman (Fred Hutchinson Cancer Research Center	N/A
pcDNA human TRIM22	Dianne Lou	CU, Boulder, CO, USA
Langerin expression plasmid	Sino Biological	HG13040-UT
DC-SIGN expression plasmid	Sino Biological	HG10200-UT
Constructs expressing LGTV NS2B/3	Previously described in Taylor et al., 2011	N/A
Constructs expressing LGTV NS5	Previously described in Taylor et al., 2011	N/A
Constructs expressing WNV NS2B/3	Previously described in Taylor et al., 2011	N/A
Constructs expressing WNV NS5	Previously described in Taylor et al., 2011	N/A
Lentiviral vectors coding shRNA (Control)	Gifted by Prof. Ralf Bartenschlager, University of Heidelberg, Germany	N/A
Lentiviral vectors coding shRNA (CypA)	Gifted by Prof. Ralf Bartenschlager, University of Heidelberg, Germany	N/A
Lentiviral vectors coding shRNA (CypB)	Gifted by Prof. Ralf Bartenschlager, University of Heidelberg, Germany	N/A
Software and Algorithms		
Prism 7 for Mac OS X	GraphPad software	https://www.graphpad.com
ZEN Imaging Software	N/A	https://www.zeiss.com/microscopy/us/products/microscope-software/zen-lite.html
ImageJ for quantifying western blots	ImageJ	https://imagej.nih.gov/ij/index.html
Flowjo for flow-cytometry	Flowjo	Licensed Software

## Highlights

- Human and rhesus macaque TRIM5α suppresses replication of specific flaviviruses
- TRIM5α restricts tick-borne but not mosquito-borne flaviviruses
- TRIM5α binds to the viral protease to promote its proteasomal degradation
- TRIM5α restricts replication of sensitive flaviviruses in human DCs
