# Supplementary material for: TRIM5α Restricts Flavivirus Replication by Targeting the Viral Protease for Proteasomal Degradation
Source: Cell Rep. Author manuscript; Available in PMC 2021 Dec 12. (PMC8666140; doi:10.1016/j.celrep.2019.05.040)
Supplement: 1 [file NIHMS1761550-supplement-1.pdf]

Cell Reports, Volume *27*

Supplemental Information

TRIM5α Restricts Flavivirus Replication

by Targeting the Viral Protease

for Proteasomal Degradation

Abhilash I. Chiramel, Nicholas R. Meyerson, Kristin L. McNally, Rebecca M. Broeckel, Vanessa R. Montoya, Omayra Méndez-Solís, Shelly J. Robertson, Gail L. Sturdevant, Kirk J. Lubick, Vinod Nair, Brian H. Youseff, Robin M. Ireland, Catharine M. Bosio, Kyusik Kim, Jeremy Luban, Vanessa M. Hirsch, R. Travis Taylor, Fadila Bouamr, Sara L. Sawyer, and Sonja M. Best

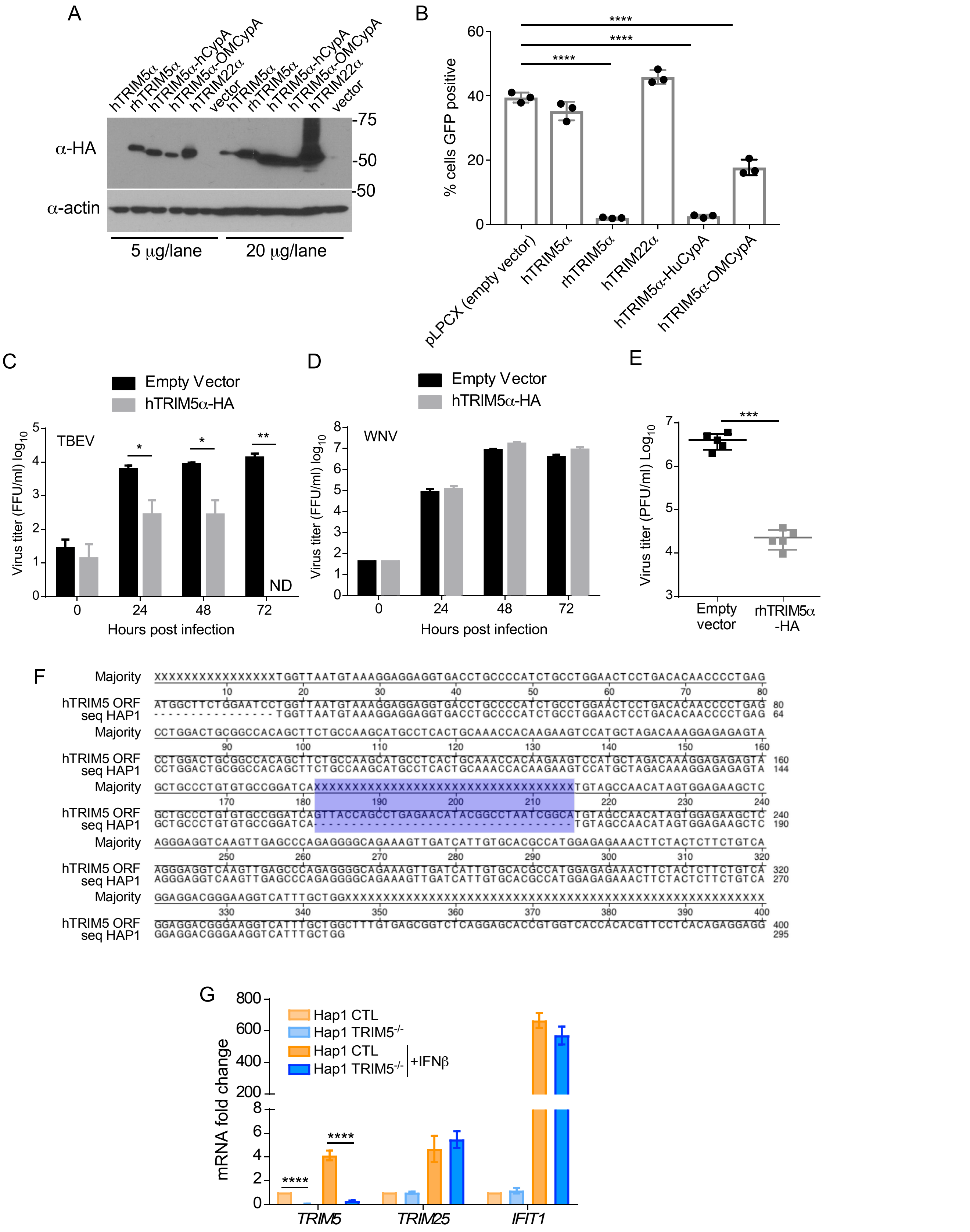

Figure S1. Human (h) and rhesus (rh) TRIM5 restrict flavivirus infection. Related to Figure 1. A. Western blot showing relative expression levels of primate TRIM5-HA in stable cell lines used in this study including hTRIM5, rhTRIM5, hTRIM5-hCypA (human cyclophilin A), hTRIM5-OMCypA (Owl monkey cyclophilin A), hTRIM22 or empty vector (control). **B.** Cell lines in A. were infected with HIV-1 virus pseudotyped with VSV-G and encoding a GFP reporter and examine for infection at 48 hpi by flow cytometry for GFP (data from 1 experiment performed in triplicates, mean ± s.d.; ****P < 0.0001, one-way ANOVA, Dunnett's post-test). **C-D.** CrFK cells stably expressing hTRIM5-HA or empty vector (control) were infected with **C.** TBEV or **D.** WNV at an MOI of 1 and infectious virus was assayed in supernatants by plaque assay at 24 h intervals. ND, none detected. Data shown from one of two experiments performed in triplicate (mean ± s.d.; *P < 0.05, **P < 0.01, Mann-Whitney U test). **E.** Titers of LGTV at 48 hpi in empty vector- and rhTRIM5α-HA HEK293 cells following infection at MOI 10 (Data from 1 experiment with 5 data points, mean ± s.d., ***P<0.001, Mann-Whitney U test). **F.** Sequencing results of the TRIM5 mRNA sequence from Hap1 cells transduced with CRISPR guide sequence for TRIM5 and Cas9. The resulting cells had a deletion in nucleotides 182-215 of the mRNA resulting in a missense mutation. **G.** mRNA expression of TRIM5, TRIM25 and IFIT1 in Hap1 gene edited to knock-out TRIM5 expression (TRIM5$^{-/-}$) and treated with IFNβ for 6 h (data from 3 experiments performed in triplicates; mean ± s.d.; *P < 0.0001, by one-way ANOVA with Tukey post-test).

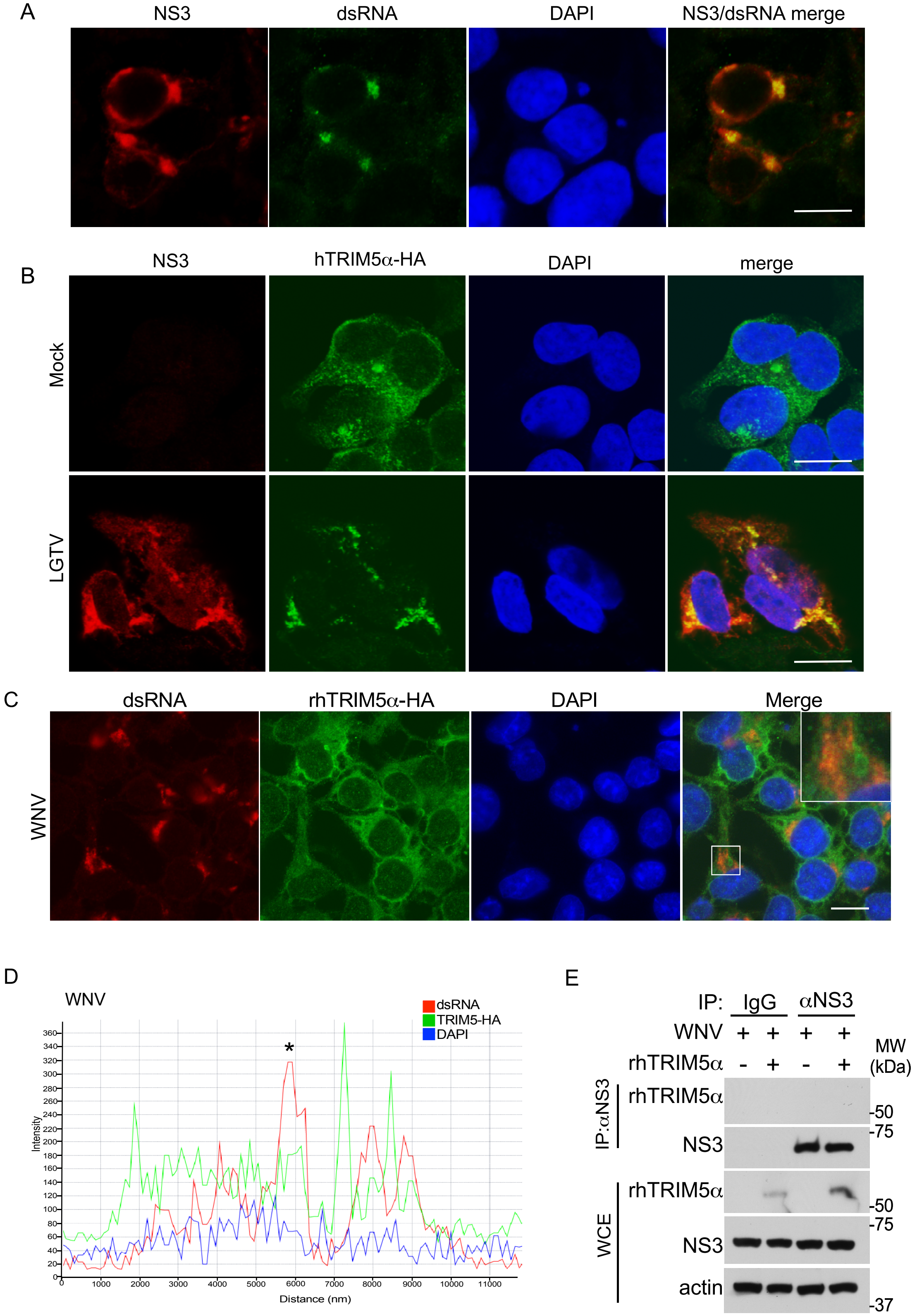

Figure S2. TRIM5 localizes to sites of viral RNA replication to mediate restriction. Related to Figure 3. A. Localization of NS3 (red) and dsRNA (green) in LGTV-infected cells. Scale bar=10μm. **B.** Localization of NS3 (red) and hTRIM5α (green) in mock- or LGTV-infected hTRIM5α-HA-expressing HEK293 cells. Nuclei were stained with DAPI (blue). Images were captured and analyzed using confocal microscopy. Scale bar=10μm. **C.** Stable rhTRIM5α-HA cells were infected with WNV (MOI 0.01) and stained for dsRNA (red) and rhTRIM5α (green) at 24 hpi. Nuclei were counterstained with DAPI. Inset shows the region indicated by a white box. Scale bar=10μm. **D.** Example of the intensity profile along vectors drawn through dsRNA staining in rhTRIM5α-HA cells infected with WNV. Asterisk indicates region of co-staining. **E.** rhTRIM5α-HA-expressing HEK293 cells were infected with WNV (MOI 0.01) and NS3 was precipitated at 48 hpi. No interacting rhTRIM5α-HA was detected.

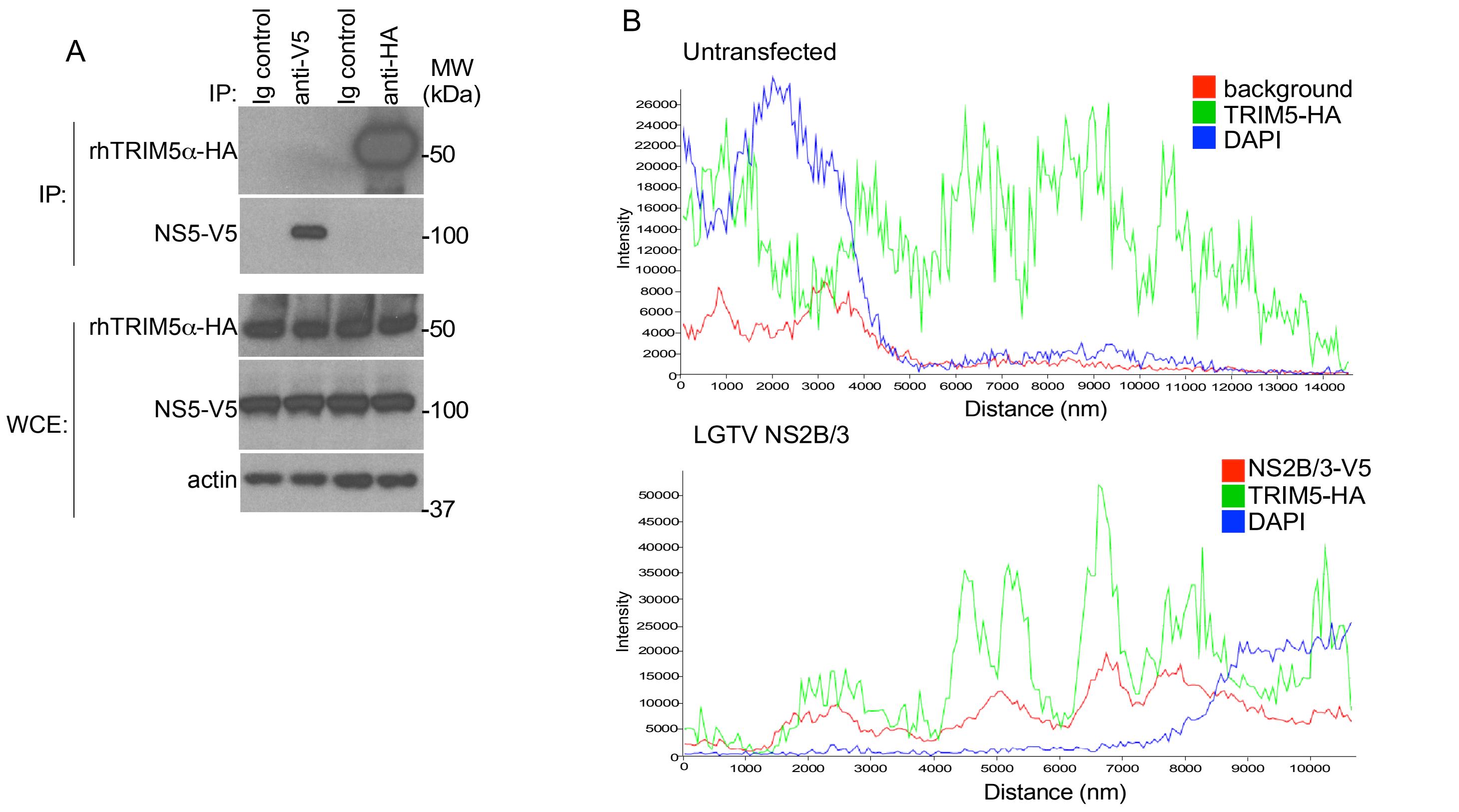

Figure S3. TRIM5α does not strongly associate with LGTV NS5. Related to Figures 3 and 5. A. Reciprocal IP of LGTV NS5-V5 or rhTRIM5α-HA following co-expression in HEK293 cells. **B.** An example of the quantification of rhTRIM5α-HA aggregation measured by intensity across vectors drawn in stable rhTRIM5α-HA HEK393 cells that are untransfected (control) or in cells positive for LGTV NS2B/3-V5. The quantification for these images is summarized in Figure 5B.

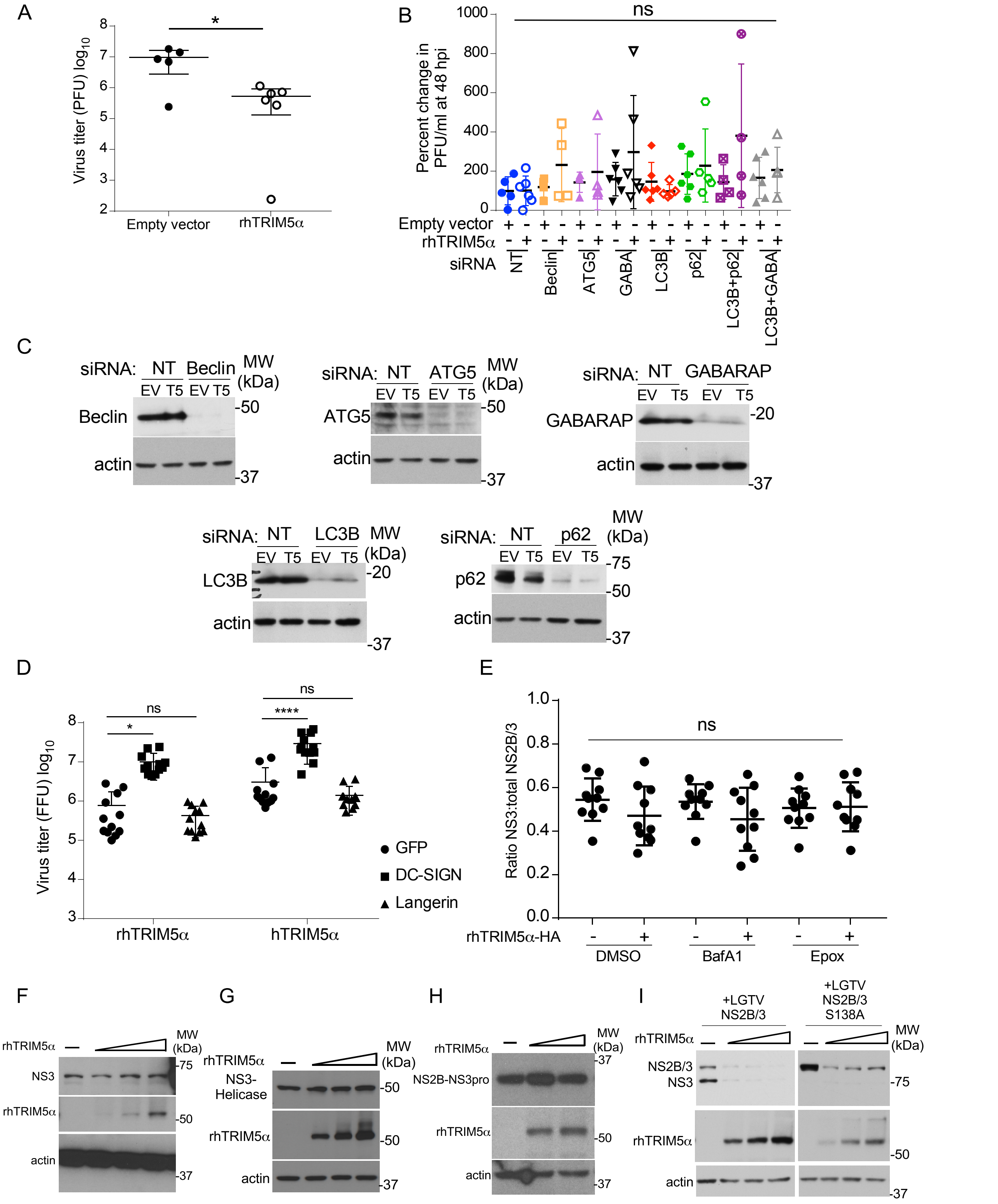

Figure S4. TRIM5 does not serve as a selective autophagy receptor during flavivirus replication. Related to Figure 5. A. LGTV titers in control and rhTRIM5-HA stable cell lines transfected with control non-targeting (NT) siRNA to demonstrate levels of restriction in these experiments without data normalization (mean ± s.d., *P < 0.05, Mann-Whitney U test). **B.** Empty vector (control) and rhTRIM5-HA stable expression cells were depleted for autophagy-related genes using specific siRNAs as indicated. At 48 h post silencing, cells were infected with LGTV (MOI 0.01) for another 48 h and virus titers from supernatants were determined using plaque assays. Data was normalized to percentage change in titer from cells treated with NT siRNA. All data are from 4-6 independent experiments performed in triplicate (ns, not significant, one-way ANOVA with Sidak post-test, mean ± s.d.). **C.** Western blot analysis for validation of siRNA knockdown 96 h post silencing using specific antibodies for BECLIN-1, ATG5, GABARAP, LC3B, p62 and b-actin in empty vector (EV) or rhTRIM5-HA (T5) cell lines. **D.** rhTRIM5-HA or hTRIM5-HA stable cell lines were transfected with expression plasmids for GFP, DC-SIGN or Langerin. 24 h later, cells were infected with LGTV (MOI of 0.01) and virus titers were determined at 48 hpi by plaque assay. Data are shown from 3 independent experiments performed in triplicate (mean ± s.d., *P < 0.05, **** P < 0.0001, ns, not significant, Mann-Whitney U test). **E.** Quantification by densitometry of the ratio of cleaved LGTV NS3: total NS2B/3 in the presence or absence of rhTRIM5α-HA across 10 independent experiments, suggesting no effect of TRIM5α on the protease activity of NS2B/3 (one-way ANOVA with Dunnett's post-test, mean ± s.d.). **F-I.** Western blot analysis of HEK293 cells transfected with increasing amounts of rhTRIM5-HA and constant amounts of **F.** LGTV NS3-V5, **G.** LGTV NS3 helicase domain, **H.** LGTV NS2B plus the NS3 protease domain, **I.** LGTV NS2B/3 S138A protease mutant. Lysates were probed specifically for HA, V5 and b-actin.

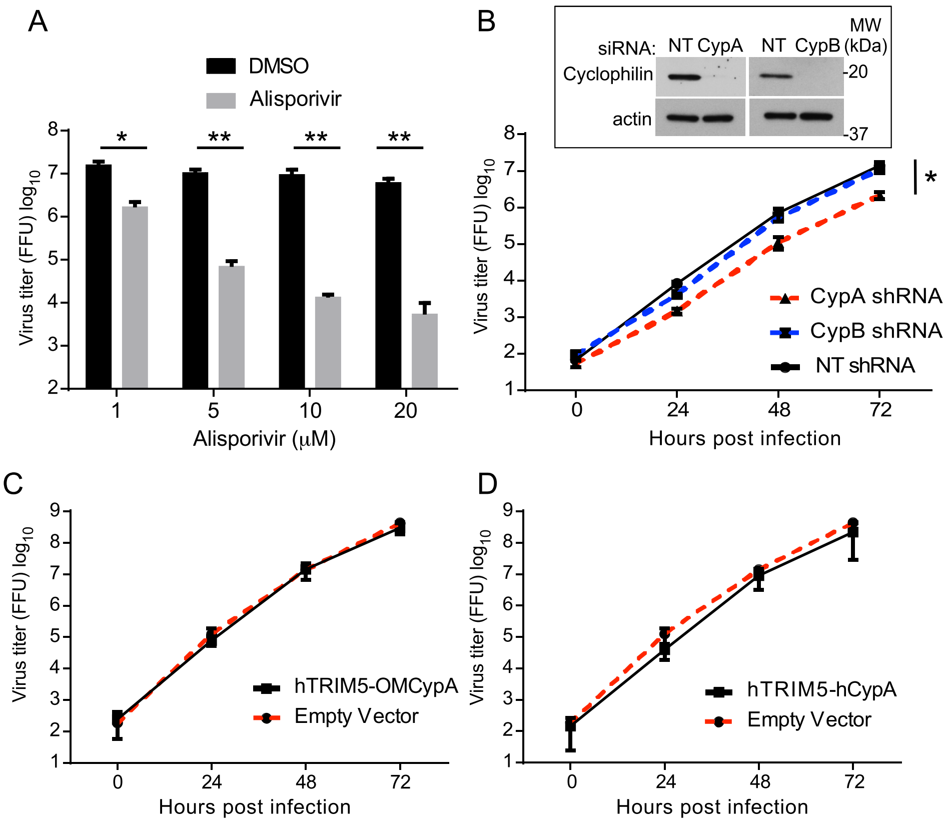

Figure S5. TRIM5 restriction of flaviviruses is independent of its association with CypA. Related to Figure 5. A. A549 cells were infected with LGTV (MOI of 0.01) for 24 h followed by treatment with increasing amounts of alisporivir or DMSO (vehicle) for 48 h. Supernatants were harvested and virus titer determined using plaque assays (data from 2 independent experiments performed in triplicate; mean ± s.d., *P<0.05; **P<0.01, Mann-Whitney U test). **B.** shRNA specific for cyclophilin A (CypA), cyclophilin B (CypB) or non-targeting (NT) control were transduced into A549 cells followed by LGTV infection (MOI of 0.01). Supernatants were harvested at indicated time points and virus titers were determined using plaque assay. Western blot inset shows specific knock down of CypA and CypB as compared to NT control. **C-D.** HEK293 cells stably overexpressing C-terminally HA tagged fusion constructs for **C.** hTRIM5-OMCypA-HA or **D.** hTRIM5-hCypA-HA were infected with LGTV (MOI of 0.01) and virus supernatants were harvested at indicated time points to determine virus titers by plaque assays (data is shown from one of two experiments performed in triplicate; mean ± s.d., *P<0.05; **P<0.01, Mann-Whitney U test).

SUPPLEMENTAL TABLES

Table S1: TaqMan probes to determine mRNA transcript levels for specific genes, related to Figure 2A and Figure S1G.

REAGENT or RESOURCE	SOURCE	IDENTIFIER
TaqMan Probes for qRT-PCR		
TRIM5	Applied Biosystems	Hs01552559_m1
HPRT	Applied Biosystems	Hs01003267_m1
IFNβ1	Applied Biosystems	Hs01077958_s1
RSAD2	Applied Biosystems	Hs00369813_m1
IL6	Applied Biosystems	Hs00985639_m1
TNFα	Applied Biosystems	Hs9999043_m1
CXCL10	Applied Biosystems	Hs0112425_g1

Table S2: siRNAs used to knockdown specific genes, related to Figure 2 and Figure S4B.

REAGENT or RESOURCE	SOURCE	IDENTIFIER
Oligonucleotides (siRNA)		
Non-Targeting	Horizon (Dharmacon)	D-001810-10-05
TRIM5	Horizon (Dharmacon)	L-007100-00-0005
Beclin-1	Horizon (Dharmacon)	L-010552-00-0005
ATG5	Horizon (Dharmacon)	L-004374-00-0005
GABARAP	Horizon (Dharmacon)	L-012368-00-0005
MAP1LC3B	Horizon (Dharmacon)	L-012846-00-0005

Table S3: Primer sequences used to generated mutations and fusion constructs for TRIM5 related to Figure 6 and S5C and D.

Oligo nucleotide information related to STAR Methods: TRIM5 Constructs-all contain C-terminal HA tag		
**List of primers used in this study**		
**Construct**	**Forward Primer**	**Reverse Primer**
human TRIM5 C15/18A RING mutations	AAGGAGGAGGTGACCGCCCCCATCGCCCTGGAACTCCTGACACAACCCCT	TGTCAGGAGTTCCAGGGCGATGGGGGCGGTCACCTCCTCCTTTACATTAA
rhesus TRIM5 C15/18A RING mutations	AAGGAGGAGGTGACCGCTCCCATCGCCCTGGAACTCCTGACAGAACCCCT	TGTCAGGAGTTCCAGGGCGATGGGAGCGGTCACCTCCTCCTTTACATTAA

Human TRIM5 up to S309 with C-term human CypA overlap	GTGCCACCATGGCTTCTGGAATCCTGGTTAATGTAAAG	GAACACGGTGGGGTTGACCATTGAAATGTTGTTTGGAGCCACTGTCAC
Human TRIM5 up to S309 with C-term owl monkey CypA overlap	GTGCCACCATGGCTTCTGGAATCCTGGTTAATGTAAAG	AATGTCGAAGAACACAGTAGGATTGACCATTGAAATGTTGTTTGGAGCCACTGTCAC
Human TRIM5delB30.2 (aa1-276)	GTGCCACCATGGCTTCTGGAATCCTGCTTAATGTAAAG	CAAGCGTAGTCTGGGACGTCGTATGGGTAGCCGCCAAACACTCTCCTTTGATTTTTTGGAAAAGTTTCTGG
Rhesus TRIM5delB30.2 (aa1-278)	GTGCCACCATGGCTTCTGGAATCCTGCTTAATGTAAAG	TCAAGCGTAGTCTGGGACGTCGTATGGGTAGCCGCCAAACACTCTCCTTTGATTTTTGTGAAAAGTTTTTGGC
